# Theoretical Framework for Predicting Dynamic Covalent Exchange Based on Proton Affinities and Proton‐Transfer Assistance

**DOI:** 10.1002/chem.71403

**Published:** 2026-07-22

**Authors:** Siebe Lekanne Deprez, Stefan J. D. Maessen, Angelina N. van Dam, Celine Nieuwland, Pascal Vermeeren, Anja R. A. Palmans, Célia Fonseca Guerra

**Affiliations:** ^1^ Department of Chemistry and Pharmaceutical Sciences, AIMMS Vrije Universiteit Amsterdam Amsterdam The Netherlands; ^2^ Department of Chemical Engineering & Chemistry and Institute for Complex Molecular Systems Eindhoven University of Technology Eindhoven The Netherlands

**Keywords:** acylsemicarbazide, density functional calculations, dynamic covalent network, energy decomposition analysis, hydrogen bonds, kinetics, supramolecular chemistry

## Abstract

We provide design principles for predicting bond exchange kinetics in acylsemicarbazide (ASC)‐based systems that can be applied to tuning the properties of dynamic networks. Because of their capability of dynamic and reversible bond dissociation, ASCs are promising motifs in the design of tunable dynamic covalent networks that combine mechanical robustness with reprocessability and stability. We elucidate the factors that determine the rate of bond dissociation using density functional theory in combination with detailed kinetic studies on the mechanism of the ASC dissociation. Several ASC compounds were investigated, R^1^─C(═O)(H)N─N(H)─C(═O)NH─R^2^, with R^1^ methyl or phenyl, and R^2^ methyl, phenyl, or benzoyl, that dissociate into hydrazide and isocyanate parts. The experimentally measured dissociation rate correlates with the proton affinity of the N─H bond next to R^2^, which could also be used to predict relative dissociation rates *a priori*. Proton‐transfer assistance is required for efficient bond exchange. A water molecule, but also neighboring ASCs (reactant) and hydrazides (product), lowers the activation barrier for bond dissociation considerably, likely facilitating autocatalysis that can occur in polymeric ASC networks. These findings can aid in the rational design of reversible polymers based on ASC motifs and can also be generalized for other dynamic covalent networks.

## Graphical abstract

Proton‑transfer catalysis controls acylsemicarbazide dissociation, linking proton affinity to exchange kinetics and enabling predictive design of robust, tunable dynamic covalent networks.

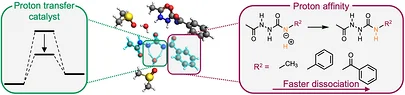

## 1 Introduction

Dynamic covalent networks (DCNs) are cross‐linked polymer materials with dynamic covalent bonds that reversibly break and reform by applying specific stimuli such as heat, light, or a catalyst [1, 2, 3, 4]. As a result, the network can change its topology, enabling properties such as reprocessability, self‐healing, and recyclability. For this reason, DCNs bridge the gap between traditional thermosets and thermoplastics by combining mechanical robustness of thermosets with dynamic adaptability of thermoplastics.

Earlier mechanistic studies of dynamic covalent bonds have demonstrated that environmental effects such as catalysis and hydrogen bonding are often essential to achieve self‐healing and reversible material behavior [5, 6, 7, 8, 9]. Because dynamic covalent networks rely on bond exchange processes with sufficiently low activation barriers, the presence of catalytic species can be required in enabling reversibility. For example, a mechanistic DFT study on anhydride‐ exchange reactions revealed that inclusion of a Brønsted acid catalyst dramatically lowers the exchange barrier from 44.1–52.8 to 25.9–33.0 kcal mol^−1^ over the temperature range 25°C–200°C [5]. This reduction in barrier height was shown to enable self‐healing, shape‐memory behavior, and composite formation. Similarly, studies on aromatic and aliphatic disulfide vitrimers demonstrated that self‐healing arises from a [2 + 1] radical‐mediated metathesis mechanism with nucleophiles acting as potential catalysts to further enhance bond reversibility [6, 8].

A promising dynamic covalent bond is the acylsemicarbazide (**ASC**) motif formed by reacting a hydrazide (R^1^─C(═O)─N(H)NH_2_) with an isocyanate (R^2^─NC(═O)) to form R^1^─C(═O)─(H)N─N(H)─C(═O)NH─R^2^ (abbreviated as **R^1^ASCR^2^**). This bond combines dynamic covalent reversibility with multiple hydrogen‐bonding sites [10, 11]. An earlier study postulated that dissociation of the dynamic covalent (N─)N**···**C(═O) bond proceeds via a dissociative pathway involving a proton transfer from the isocyanate to the hydrazide moiety and a simultaneously C─N bond cleaving, passing through a four‐membered transition state (see Scheme 1a) [11]. The authors further suggested that, in polymeric environments, hydrogen‐bonded networks could substantially lower the dissociation barrier by facilitating bond cleavage. However, this hypothesis is based on molecular dynamics conformational scans, without exploration of the full potential energy surface. Moreover, solvent effects and alternative **R^1^ASCR^2^** compounds (i.e., with different substituents) were not considered.

**SCHEME 1:**
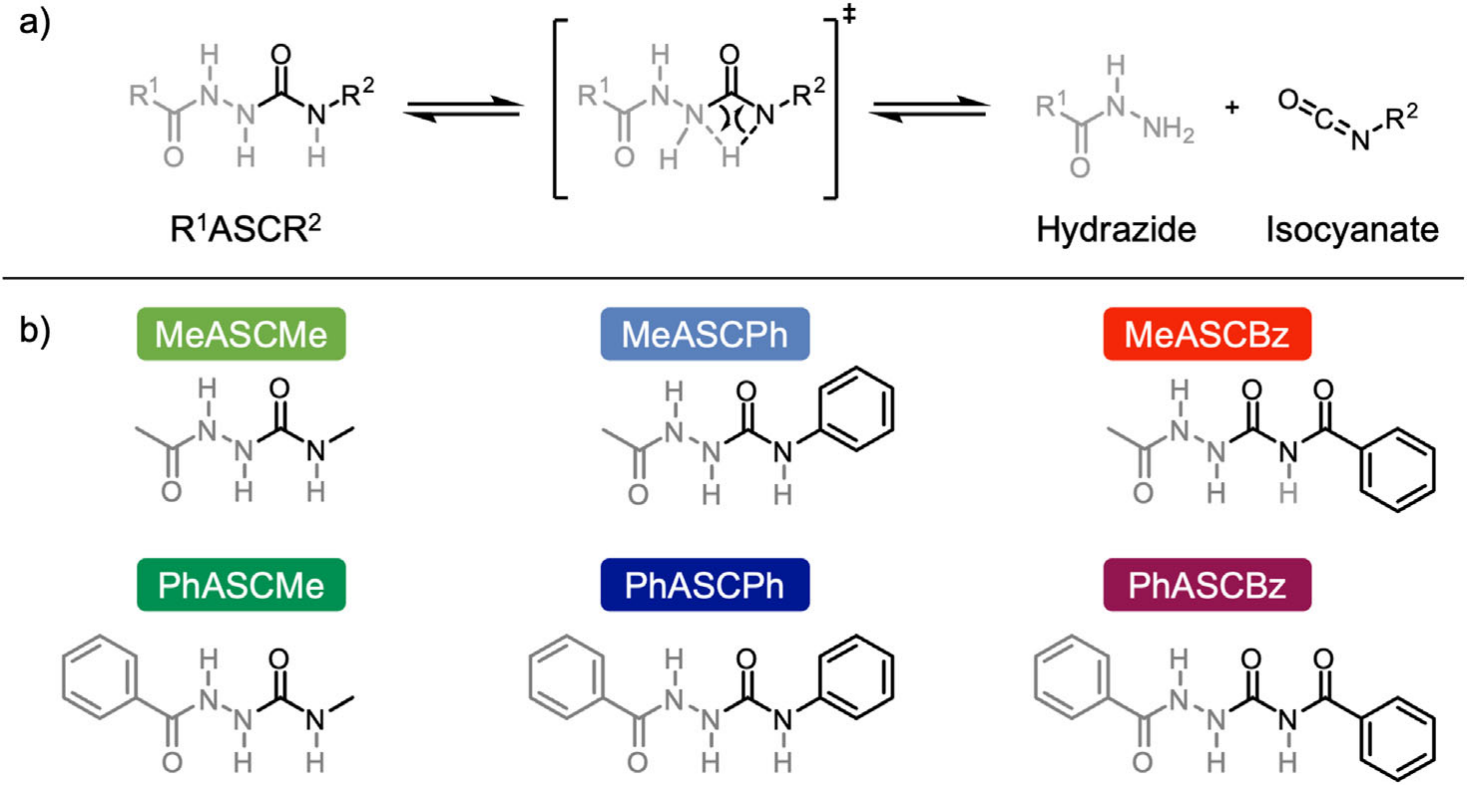
(a) ASC dissociation mechanism proposed in literature characterized by a four‐membered ring transition state [11]. (b) ASC compounds considered in this study.

To further elucidate structure–property relationships, we previously investigated the influence of hydrazide (R^1^) and isocyanate (R^2^) substituents [10]. Measured dissociation rates revealed that variations in the R^2^ isocyanate group exert a pronounced influence on reactivity: replacing a methyl (Me) group with phenyl (Ph) and benzoyl (Bz) substantially accelerates dissociation, whereas modification of the R^1^ hydrazide substituent has a considerably smaller effect. We hypothesized that these differences arise from electronic stabilization provided by the substituents [10]. Since the N─H bond cleavage in the transition state resembles a deprotonation process, yielding a (partial) anionic species, the conjugated phenyl and benzoyl groups may stabilize this transition state by enhancing delocalization and thereby increasing the dissociation rate. Nevertheless, the dissociation mechanism remains incompletely understood, and the origin of the substituent effects (particularly for R^2^) is not fully resolved. Given that **ASC** motifs combine dynamic covalent bonding with intrinsic hydrogen‐bonding capabilities, catalytic or proton‐transfer processes may play a critical role in governing bond exchange. Consequently, further experimental and computational studies are required to establish a comprehensive mechanistic picture.

In this study, we present a combined experimental and computational mechanistic investigation aimed at elucidating how solvation, substituent effects, and proton‐transfer catalysis control the dissociation behavior of **R^1^ASCR^2^** moieties with R^1^ = methyl (Me), phenyl (Ph), and R^2^ = Me, Ph, and benzoyl (Bz). To this end, we perform density functional theory (DFT) computations (see Section S1 for computational details) and we utilize quantitative Kohn–Sham molecular orbital (KS‐MO) [12, 13] theory along with a complementary energy decomposition analysis (EDA) [14, 15] scheme to provide a mechanistic foundation for rationalizing and predicting bond‐exchange kinetics in **ASC**‐based systems, offering concrete design principles for the development of self‐healing materials. We explain the difference in dissociation rates observed for various **R^1^ASCR^2^** moieties through their relationship with proton affinities. Moreover, we examine how dissociation barriers are modulated by solvent and by the presence of proton‐transfer catalysts, supported by direct experimental validation.

## 2 Results and Discussion

### 2.1 Proton Affinity Correlates With the ASC Dissociation Rate

To systematically study the influence of the substituents on the dynamic character of the N─H bond, we consider the following **R^1^ASCR^2^** model systems: **MeASCMe**, **MeASCPh**, **MeASCBz**, and **PhASCMe**, **PhASCPh**, and **PhASCBz** (see Scheme 1b). The postulated dissociation mechanism (see Scheme 1a) shows that the N─H^a^, the N─H bond that is part of the isocyanate moiety, is cleaved, followed by a transfer of H^a(+)^ to the hydrazide moiety [11]. We hypothesize that the rate of **ASC** dissociation depends on the strength of the N─H bond; a weaker bond may facilitate faster dissociation. **ASC** bond dissociation rates (*k*_d_) were determined from in situ ^1^H NMR experiments conducted in DMSO‐d*_6_* over the temperature range 70°C–130°C, assuming pseudo‐first‐order kinetics as established in previous studies (see Figure 1a) [10, 11]. To prevent recombination (i.e., making the reaction irreversible), benzylamine was added to act as a trapping agent for the isocyanate product (O═C═N─R^2^, see Section S3.5 for experimental methods).

**FIGURE 1:**
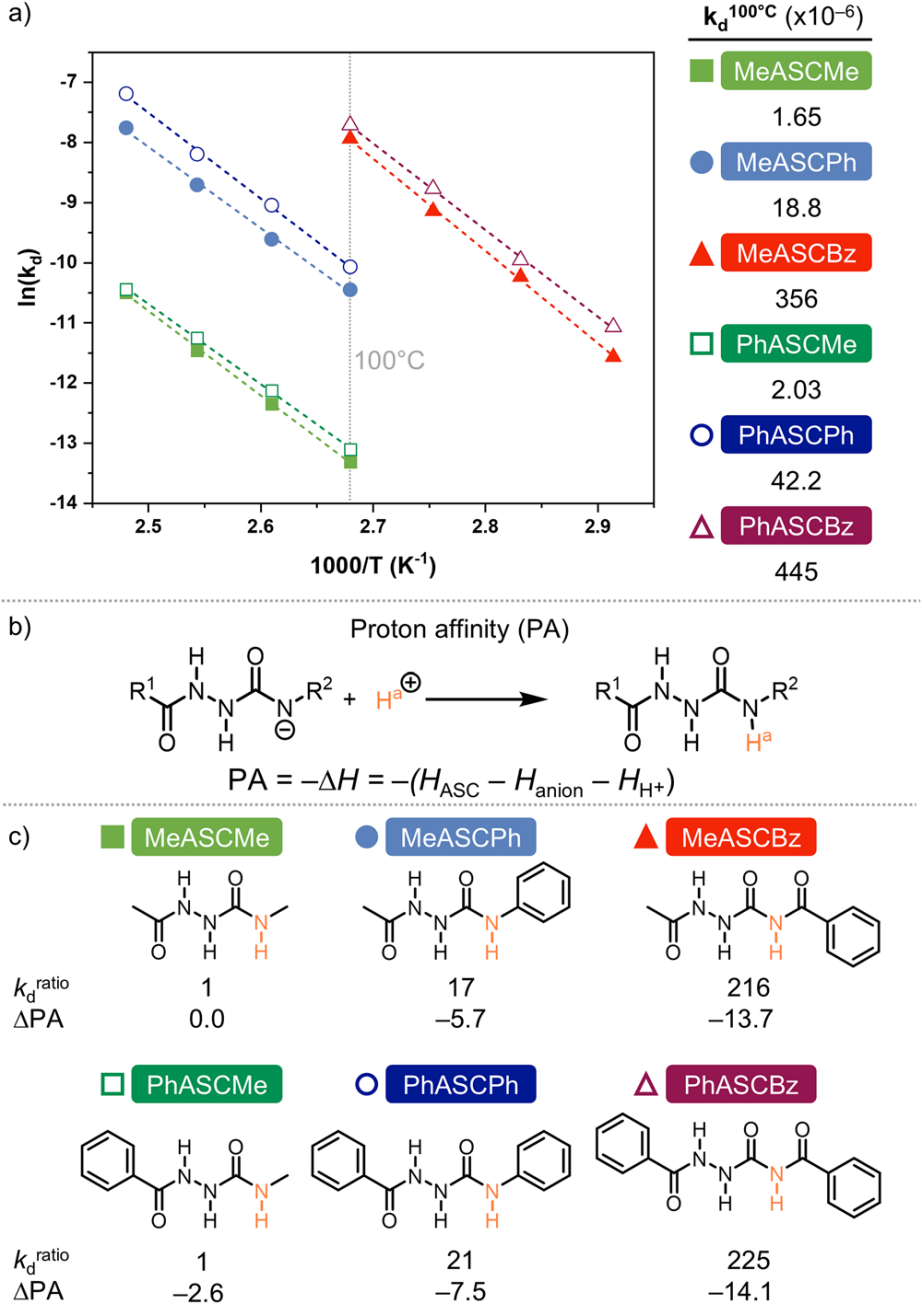
(a) Arrhenius plot of the experimental R^1^ASCR^2^ dissociation rates *k*_d_ (in s^−1^) with the vertical line indicating the rates at 100°C, derived from ^1^H NMR in DMSO‐*d_6_*. (b) Reaction scheme and definition of proton affinity. (c) Dissociation rates (at 100°C) and relative proton affinities (ΔPA in kcal mol^−1^) with respect to MeASCMe and PhASCMe, computed at ZORA‐BLYP‐D3(BJ)/TZ2P.

The results show that the dissociation rate increases systematically when R^2^ changes from Me to Ph to Bz. **MeASCPh** and **MeASCBz** dissociate, respectively, 17 and 216 times faster than **MeASCMe**, and **PhASCPh** and **PhASCBz** dissociate 21 and 225 times faster than **PhASCMe** (see Figure 1c). Going from **MeASCR^2^** to **PhASCR^2^**, that is, having the same R^2^ substituent but different R^1^ substituents, shows a smaller increase in the dissociation rate. Thus, varying R^2^ exerts the strongest influence on the rate while the R^1^ substituent exerts a weaker effect. We postulate that the stronger influence of R^2^ might be because it is directly attached to the active proton dissociation center N(─H^a^)─R^2^ [10].

To investigate the correlation between varying the substituents and the trend in dissociation rates, we evaluated the proton affinity (PA) of the N─H^a^ bond. This is the negative of the enthalpy change associated with heterolytic formation of the N─H^a^ bond from the conjugate base (anion) and proton H^+^ in the gas phase (see Figure 1b) [16]. The PA represents an intrinsic measure of the N─H^a^ bond strength and is therefore solvent‐independent. It is closely related to acidity, an extrinsic property quantified by the p*K*_a_, which is calculated from the negative Gibbs free energy change in the presence of a solvent, often water. More positive PA and p*K*_a_ values indicate a stronger bond (i.e., more basic), while more negative or lower values indicate weaker bonds (i.e., more acidic).

In Figure 1c, we report the relative PA values, ΔPA, of **R^1^ASCR^2^** monomers with respect to **MeASCMe** (see Table S1 for PA values). The ΔPA values become increasingly more negative from −5.7 kcal mol^−1^ for **MeASCPh** to −13.7 kcal mol^−1^ for **MeASCBz**, and a similar trend is observed for **PhASCR^2^** series with −2.6 kcal mol^−1^ for **PhASCMe**, −7.5 kcal mol^−1^ for **PhASCPh**, and −14.1 kcal mol^−1^ for **PhASCBz**. We therefore conclude that the N─H^a^ bond becomes progressively weaker as R^2^ is varied from Me to Ph to Bz, consistent with the observed increase in dissociation rates. The proton affinity results also become more negative when changing R^1^ = Me to Ph because for each fixed R^2^, the dissociation rate increases, and the bond becomes weaker (more negative ΔPA value).

To understand why the N─H^a^ bond weakens, we performed energy decomposition analyses (EDA [14, 15], see Theoretical Methods) in the framework of quantitative KS‐MO [12, 13]. EDA allows us to decompose the interaction energy between the anion and proton, Δ*E*_interaction_, into two dominant bonding contributions (see Figure 2a): (i) the electrostatic interactions between the charge distribution of the anion and positively charged proton, Δ*V*_electrostatics_, and (ii), the orbital interactions, Δ*E*_orbital interactions_, reflecting charge donation from the 2p‐derived orbital on the N^−^ atom into the empty 1s orbital of H^+^. Notice that there is no Pauli repulsion since the proton has no occupied orbitals, thus experiencing no repulsion stemming from orbital overlap with the occupied orbitals of the anion.

**FIGURE 2:**
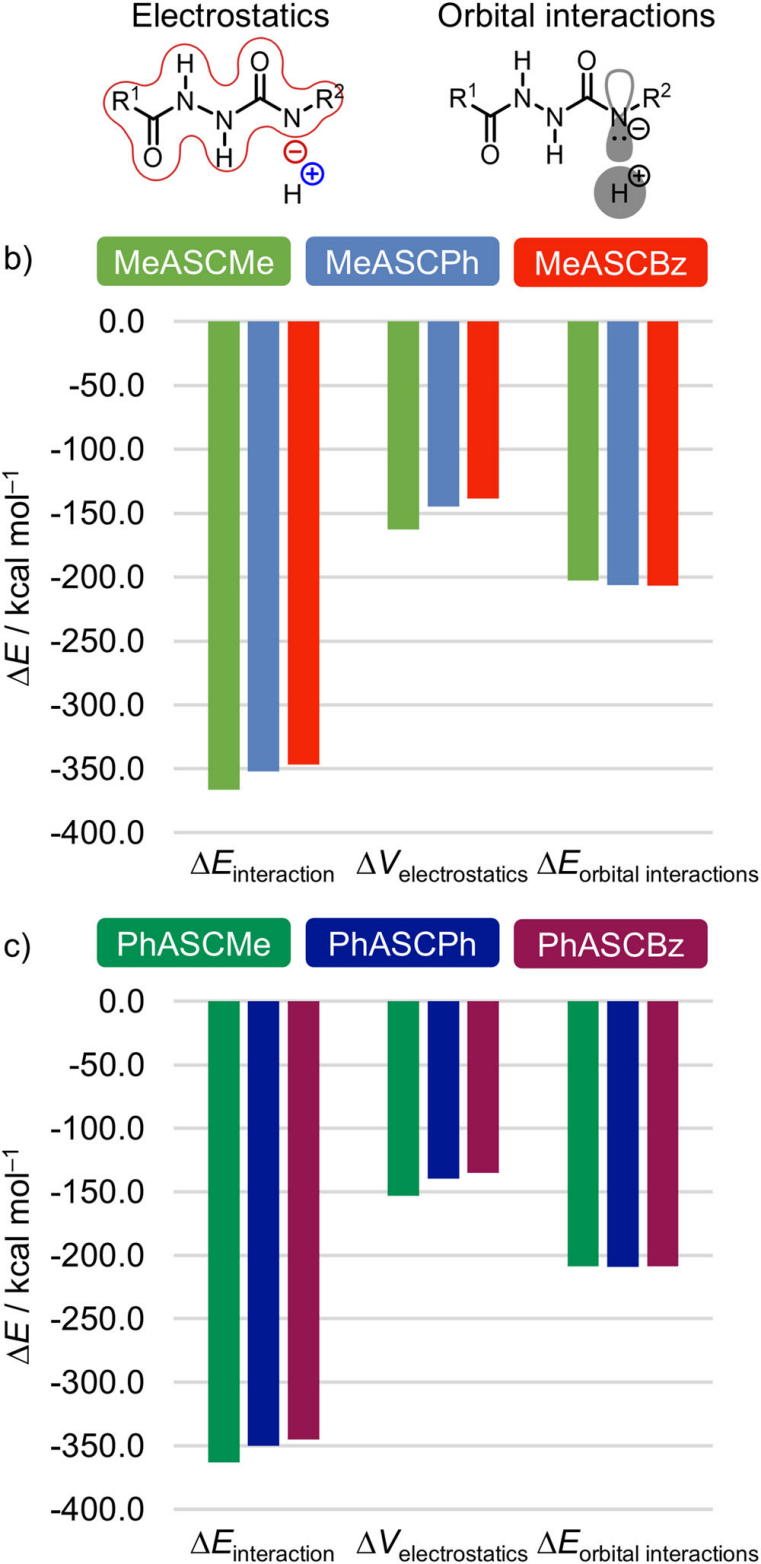
(a) Schematic visualizations of electrostatics and orbital interaction terms. (b) Energy decomposition analyses (EDAs) of the heterolytic N─H bond cleavage for MeASCR^2^ and c) PhASCR^2^ monomers with R^2^ = Me, Ph, or Bz, computed at ZORA‐BLYP‐D3(BJ)/TZ2P.

We observe from Figure 2b that Δ*E*_interaction_ becomes less negative for the **MeASCR^2^** series, indicating a weaker interaction when R^2^ changes from Me to Ph to Bz (note: contrary to PA, more negative Δ*E* values indicate stronger bonds). The same trend is observed for the **PhASCR^2^** series with R^2^ = Me, Ph, and Bz (see Figure 2c). The orbital interactions term does not explain the trend in Δ*E*_interaction_ because Δ*E*_orbital interactions_ remains essentially constant for **PhASCR^2^** or becomes even slightly more stabilizing (more negative) for **MeASCR^2^**. Therefore, the weakening of the N─H^a^ bond originates from the electrostatic term because both Δ*E*_interaction_ and Δ*V*_electrostatics_ become less negative from Me to Ph to Bz.

Further analysis in Section S2.3 shows that the trend in electrostatics is caused by the phenyl and benzoyl substituents withdrawing more electronic charge density, leading to a weaker interaction between the deprotonated N atom and the proton (see Table S4 and Figure S1). Thus, varying the R^2^ substituent in the order Me, Ph, and Bz, increases the reaction rate because the N─H^a^ bond becomes weaker, owning to the better stabilization (i.e., delocalization) of the negative charge on the conjugated base.

### 2.2 Unimolecular Dissociation

With the effect of varying the R^2^ established, we now study the effect of varying the chemical environment by including solvent (implicitly and explicitly) and by accounting for assisting molecules (e.g., catalysts) on DFT level. Our starting point is the earlier proposed mechanism involving **MeASCMe** (shown in Scheme 1a) [11]. Figure 3 shows the potential energy surfaces (PES) and transition‐state structures of the unimolecular dissociation involving **MeASCMe**, **MeASCPh**, and **MeASCBz**, in the gas phase (Figure 3a) and DMSO (Figure 3b). We show the reactant (R), reactant complex (RC, if present), product complex (PC), and products (P). Also, we report enthalpies (∆*H*) instead of Gibbs free energies (∆*G*) because the enthalpies are related to the activation energy, following from the Eyring–Polanyi equation (see Section S1.6 and Equation S17).

**FIGURE 3:**
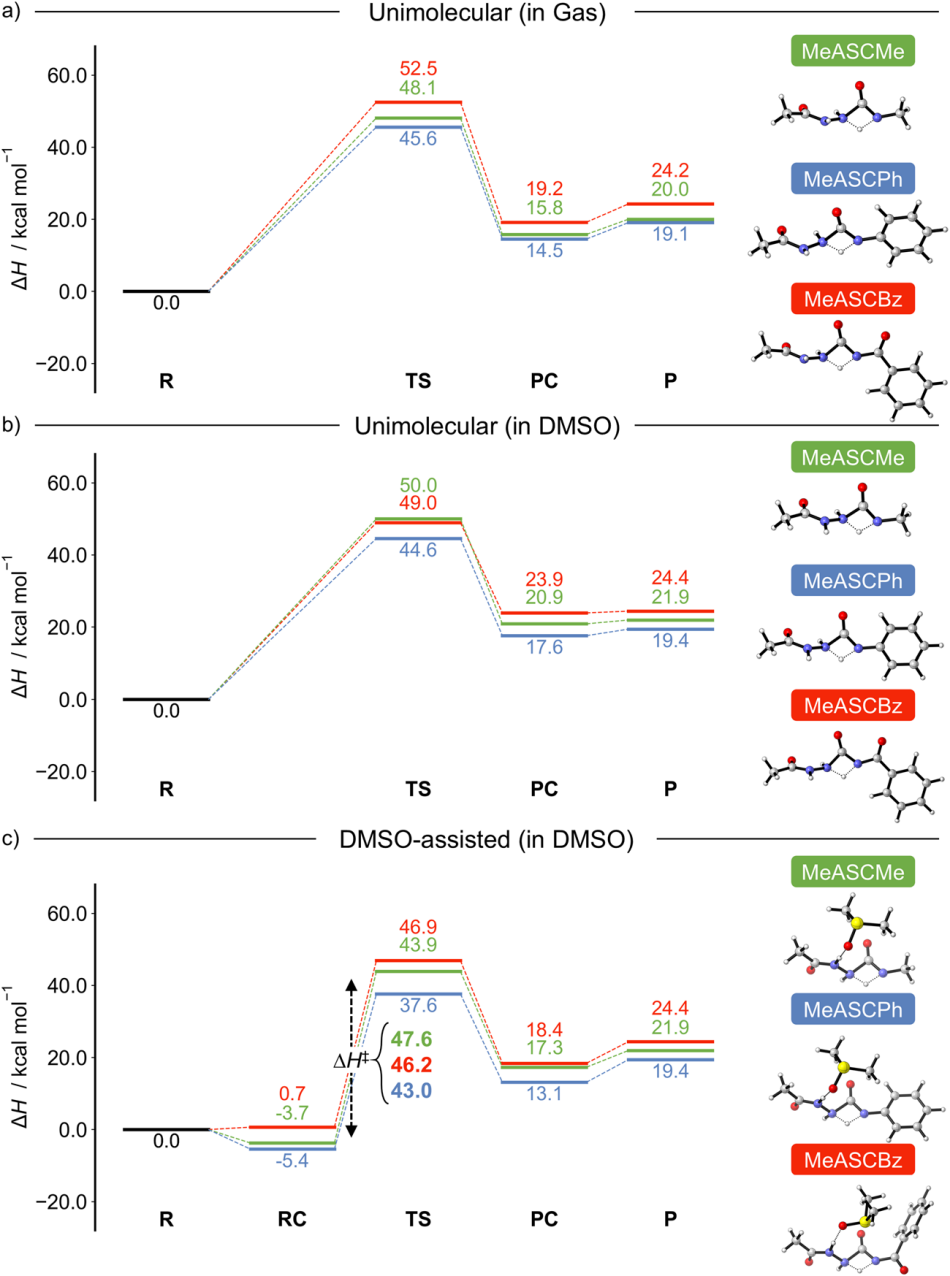
Potential energy surfaces (Δ*H*, in kcal mol^−1^) computed for the unimolecular dissociation of MeASCR^2^ monomers in (a) the gas phase, (b) DMSO, and (c) explicit DMSO. Transition‐state structures are shown next to the PES, computed at [COSMO(DMSO)]‐ZORA‐M06‐2X/TZ2P//[COSMO(DMSO)]‐ZORA‐BLYP‐D3(BJ)/TZ2P.

We find similar transition‐state structures to those proposed in the literature, characterized by the four‐membered ring‐like motif [11]. But, the dissociation barriers are substantial for the unimolecular dissociation reaction with barriers between 45.6 and 52.5 kcal mol^−1^ in gas phase and 44.6 and 50.0 kcal mol^−1^ in (implicit) DMSO. Such significant barriers are not in line with experiments and fail to reproduce the dynamic aspect of a dynamic covalent bond since we estimate activation barriers of ∼26–31 kcal mol^−1^ based on the experimental rates that are shown in Figure 1 (see Table S2). Moreover, the order of barrier heights upon varying R^2^ does not match experimental rates. We expect that R^2^ = Me proceeds with the highest dissociation barrier while R^2^ = Bz with the lowest. But, this is not observed for either the **MeASCR^2^** series (Figure 3) or the **PhASCR^2^** series (see Figure S2). Congruent with earlier work [10], we conclude that the unimolecular dissociation is not the correct mechanism regardless of (implicit) solvent effects.

### 2.3 Can DMSO Assist Dissociation?

Because the barriers associated with unimolecular dissociation are prohibitively high, it is reasonable to consider whether the solvent DMSO could facilitate the reaction by forming hydrogen bonds with **ASC** compounds and thereby stabilizing the transition state. However, we find that explicit treatment of DMSO does not meaningfully reduce the dissociation barriers, failing to illustrate the dynamic properties of the R^1^─CO─NHNH···CONH‐R^2^ bond in **ASC** compounds. The PES profiles were computed for **R^1^ASCR^2^** compounds with one explicit DMSO molecule coordinating to the reactive site (see Figure 3c and Figure S2c). The results show that DMSO does not assist the dissociation process: in all cases, the DMSO molecule does not coordinate to the active site. Instead, it forms a hydrogen bond at a different location on the **ASC** framework. Although these hydrogen‑bond interactions can slightly stabilize the transition‐state relative to the isolated reactant (R), the barriers referenced to the reactant complex (RC) remain essentially unchanged from those of the unimolecular pathway. Furthermore, DMSO‑assisted pathways predict the lowest barrier for **MeASCPh** rather than for **MeASCMe**, which contradicts experimental trends in reactivity.

To assess experimentally whether DMSO actively participates in the dissociation process, we performed kinetic experiments with **PhASCPh** and benzylamine as trapping agent (*vide supra*) in which DMSO was replaced by 1,1,2,2‐tetrachloroethane (TCE). TCE has a significantly lower dielectric constant (*ε* = 8.4) than DMSO (*ε* = 46.7) and is incapable of forming hydrogen bonds with **PhASCPh**. Figure 4 shows the temperature‐dependent dissociation rates in TCE (orange squares) and DMSO (blue circles).

**FIGURE 4:**
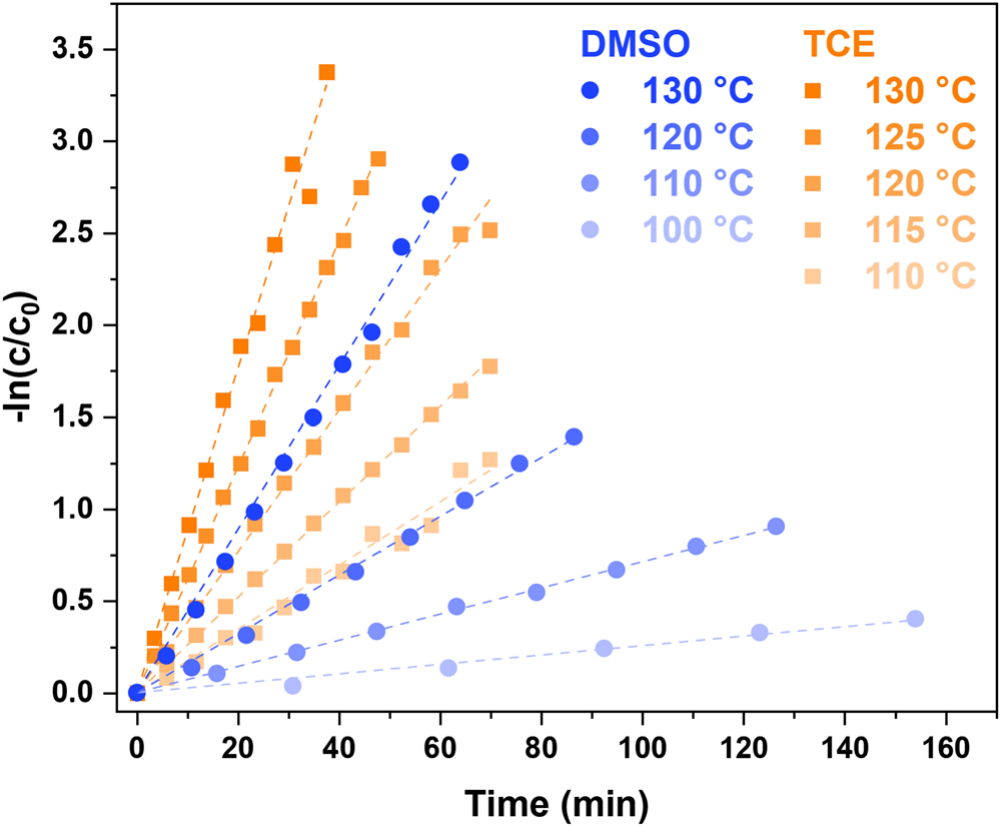
Kinetic measurements of the dissociation of PhASCPh in 1,1,2,2‐tetrachloroethane (TCE) and DMSO, measured at temperatures between 110°C–130°C.

Surprisingly, the dissociation proceeds faster in TCE, as seen by the steeper slopes of the first‐order kinetic fits. This suggests that hydrogen‐bonding interactions with DMSO do not play a dominant role in accelerating the dissociation. Instead, it is more plausible that the higher apparent dissociation rates in TCE can be attributed to localization effects. We propose that, in the less polar TCE environment, the reactants are likely to remain in closer proximity, increasing the probability that a benzylamine molecule is immediately available to trap the transient isocyanate intermediate. This would lead to a higher number of successful, observable dissociation events. In contrast, in polar DMSO the hydrazide fragment may more readily re‐attack the isocyanate to reform the same **ASC**, a process that would reduce the effective dissociation rate without being detectable by NMR spectroscopy. Additionally, aggregation or dimerization phenomena may contribute as the **ASC** concentration approaches its solubility limit in TCE under these conditions.

Regardless of the precise origin, the faster dissociation observed in TCE demonstrates that any potential stabilizing or activating role of DMSO through hydrogen bonding is unlikely to be the primary factor governing the dissociation kinetics.

### 2.4 Water‐assisted Dissociation

Because neither the unimolecular nor the “DMSO‑assisted” dissociation pathways reproduce the experimentally observed rates—both yielding activation barriers that are too high—we considered alternative mechanisms that could facilitate bond cleavage. A key step during dissociation is the transfer of the N─H^a^ proton from the isocyanate to the hydrazide side (see Scheme 1a). Any assisting molecule must therefore be both a proton acceptor (to abstract a proton from the isocyanate side) and proton donor (to donate a proton to the hydrazide side). Given the fact that DMSO is hygroscopic and readily absorbs trace amounts of water, we hypothesized that water may participate in the reaction and reduce the barrier by mediating proton transfer. Thus, we propose the water‑assisted mechanism depicted in Figure 5a.

**FIGURE 5:**
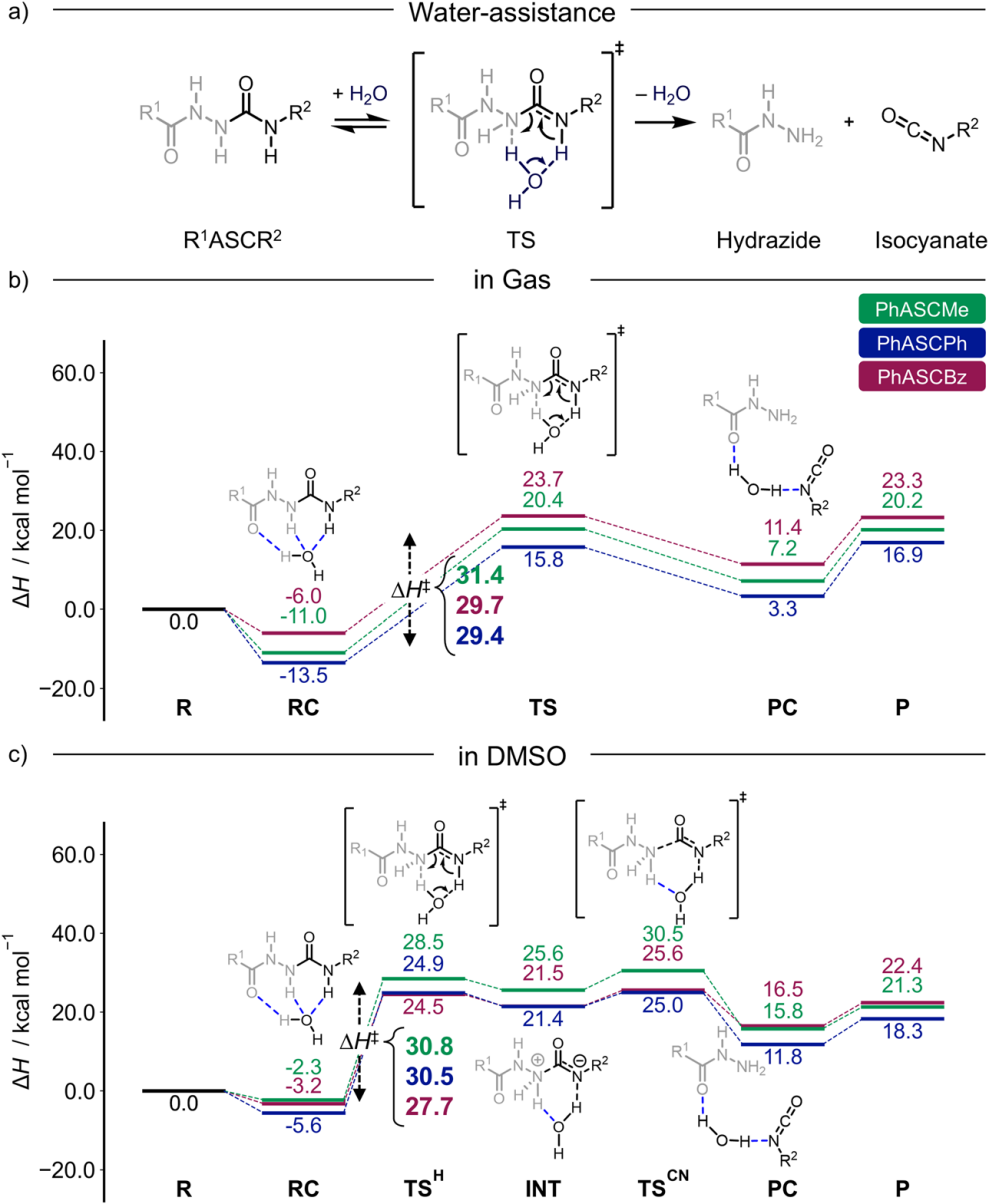
(a) Water‐assisted dissociation of R^1^ASCR^2^ involving water‐assisted proton transfer, resembling a six‐membered transition state, with R^1^ = Me, and R^2^ = Me, Ph, and Bz and potential energy surfaces (Δ*H*, in kcal mol^−1^) of the water‐assisted dissociation of PhASCMe, PhASCPh, and PhASCBz in (b) the gas phase and (c) implicit DMSO. Dashed bonds in blue indicate hydrogen bonds. Computed at [COSMO(DMSO)]‐ZORA‐M06‐2X/TZ2P//[COSMO(DMSO)]‐ZORA‐BLYP‐D3(BJ)/TZ2P.

Herein, a water molecule abstracts the (N─)H^a^ proton while simultaneously donating one of its own hydrogen atoms to the hydrazide nitrogen, resembling a six‐membered ring in the transition‐state instead of the four‐membered ring as in the unimolecular mechanism (*vide supra*). Concurrently, the N─C bond elongates and ultimately cleaves, regenerating water (i.e., serving as catalyst). We found transition states that substantially lower the dissociation barriers relative to the unimolecular pathway in the gas phase (see Figure 5b). The barriers decrease from Δ*H* ^‡^ = 43.0–47.6 kcal mol^−1^ for the unimolecular dissociation (Figure 3c) to Δ*H* ^‡^ = 29.4–31.4 kcal mol^−1^ (Figure 5b) and Δ*H* ^‡^ = 27.7–30.8 kcal mol^−1^ in implicit DMSO (Figure 5c) for the water‑assisted pathway. The water‐assisted barriers in DMSO are in excellent agreement with the estimated barriers heights of 25–30 kcal mol^−1^ (see Table S2). Along R^2^, the barriers decrease in the expected order: **PhASCMe** has the highest barrier of 30.8 kcal mol^−1^, followed by **PhASCPh** (30.5 kcal mol^−1^) and, finally, **PhASCBz** with the lowest barrier of 27.7 kcal mol^−1^, with respect to the reactant complex in implicit DMSO (see Figure 5c). This pronounced barrier‑lowering effect of water‑assisted proton transfer is consistent with previous studies on tautomerization reactions, where water participation similarly leads to substantial reductions in activation barriers [17, 18, 19, 20].

To experimentally support our computational findings, we measured the dissociation rates of **PhASCPh** in 1,1,2,2‑tetrachloroethane (TCE) and DMSO under both dry ( < 40 ppm H_2_O) and wet (∼1000 ppm H_2_O) conditions (see Table 1 and Section S3.2 for solvent preparation). In both solvents, dissociation proceeds slightly more slowly under dry conditions than under wet conditions. This trend indicates that trace amounts of water can influence the dissociation process in both polar (DMSO) and nonpolar (TCE) media. Importantly, however, the magnitude of this effect is small, suggesting that although water may assist during **ASC** dissociation, its experimental impact under these conditions is limited, which is especially unexpected in the dry solvents where the absence of water was theorized to lower the dissociation rate. These observations imply that additional mechanisms must play a substantial role in determining the effective reaction kinetics (*vide infra*).

**TABLE 1:** Dissociation rates *k*_d_ (in s^−1^) of PhASCPh in DMSO and TCE, respectively, measured at 120°C with 3 equivalences of benzylamine.

Solvent	*k*_d_ in dry conditions	*k*_d_ in wet conditions
DMSO	2.68 · 10^−4^ s^−1^	2.88 · 10^−4^ s^−1^
TCE	6.20 · 10^−4^ s^−1^	6.53 · 10^−4^ s^−1^

### 2.5 General Assistance

In the absence of water, proton transfer does not necessarily become inaccessible, as several other species present in the mixture can act as shuttles. Benzylamine, the hydrazide fragment (one of the dissociation products), and the reactant **R^1^ASCR^2^** itself all possess functional N─H groups that may facilitate the proton shuttling during dissociation. The involvement of water, therefore, reflects a more general mechanistic principle: dissociation can be assisted by any species capable of both proton donation and acceptance, with water being one example.

To demonstrate this generality, we first examined benzylamine as a potential external proton‑transfer catalyst. Previously, benzylamine's role was purely a trapping agent for the isocyanate, rendering the reaction irreversible (see Figure 6a). Now, we propose that it can additionally function as an external proton‑transfer catalyst. Figure 6b shows that the dissociation of **PhASCPh** in DMSO increases systematically with added benzylamine (1–3 equivalents), confirming that proton‑transfer assistance enhances the rate. Corresponding computational PES profiles (see Figure 6c) reveal that benzylamine lowers the activation barriers to values comparable to those obtained for the water‑assisted pathway (Δ*H* ^‡^ = 34.6 kcal mol^−1^ for **PhASCMe**, Δ*H* ^‡^ = 28.3 kcal mol^−1^ for **PhASCPh**, and Δ*H* ^‡^ = 26.2 kcal mol^−1^ for **PhASCBz**, relative to the RC), and substantially below the unimolecular barriers (Δ*H* ^‡^ = 43.0–47.6 kcal mol^−1^, see Figure 3). These combined experimental and computational results establish benzylamine as a competent proton‑transfer catalyst.

**FIGURE 6:**
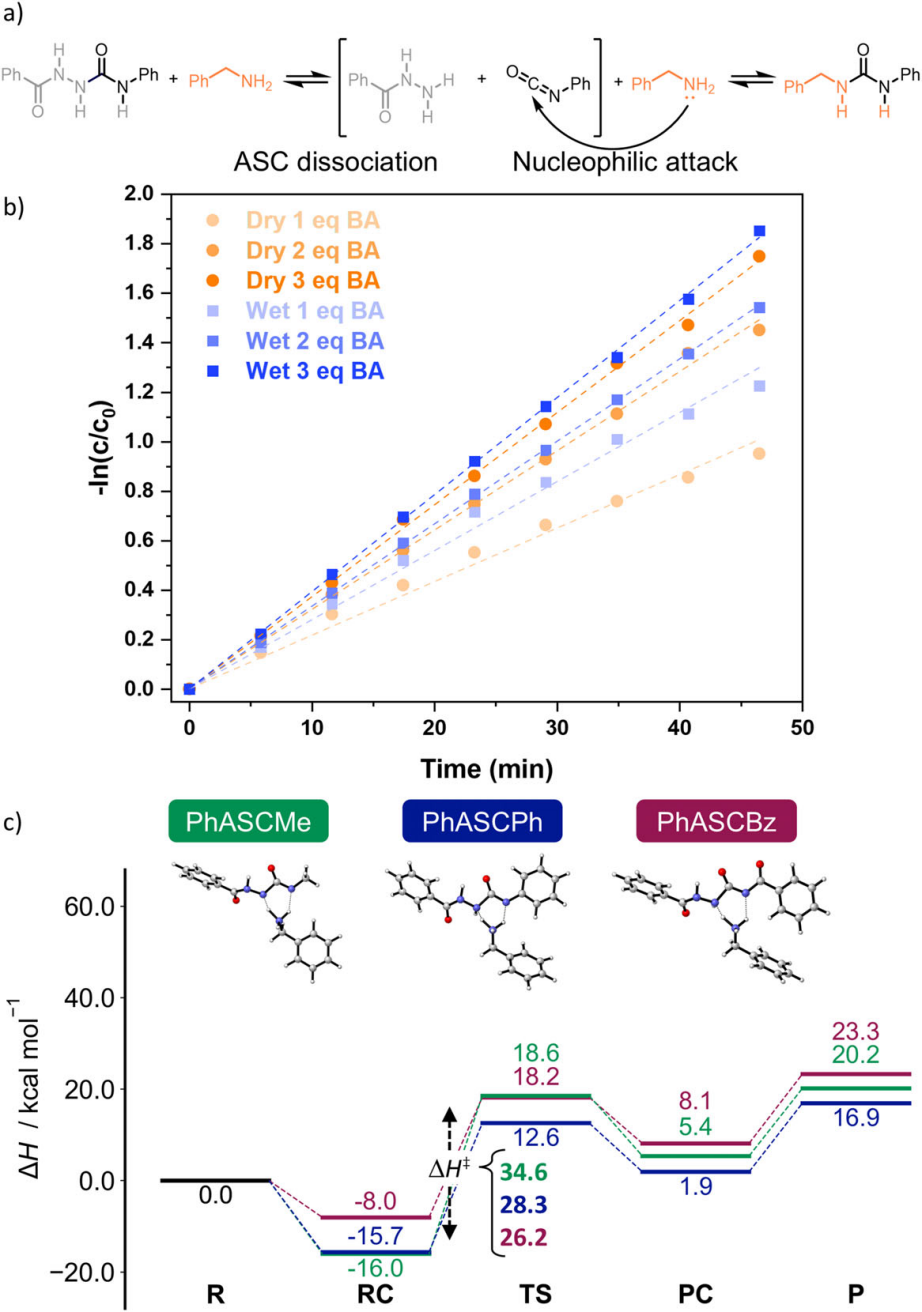
(a) Reaction scheme of the dissociation of PhASCPh with benzylamine acting as catalyst and trapping agent. (b) Kinetic measurements of PhASCPh in dry DMSO (<40 ppm H_2_O) and wet (∼1000 ppm H_2_O) DMSO (see Section S3.2), measured at 120°C. (c) Potential energy surface (Δ*H*, in kcal mol^−1^) of PhASCR^2^ with R^2^ = Me, Ph, and Bz, including their transition states in gas phase, computed at ZORA‐M06‐2X/TZ2P//ZORA‐BLYP‐D3(BJ)/TZ2P.

Finally, to further substantiate the generality of proton‑shuttle catalysis, we evaluated two additional species that are inherently present in the ASC reaction environment: the hydrazide product and a second **R^1^ASCR^2^** molecule containing an N–H functional group. Gas‑phase PES profiles for these **MeASCR^2^** show the hydrazide‐assisted (Figure 7b) and ASC‐assisted (Figure 7c) dissociation pathways with dissociation barriers comparable to the water‑assisted mechanism, and decisively lower than the unimolecular pathway. Substituting R^1^ = Me for R^2^ = Ph yields similar results (see Figure S3). In solution‐phase, we observe that the mechanisms become more stepwise and may involve additional intermediates (see Figures S4 and S5), but the barriers are equivalently lowered similar to in the gas‐phase.

**FIGURE 7:**
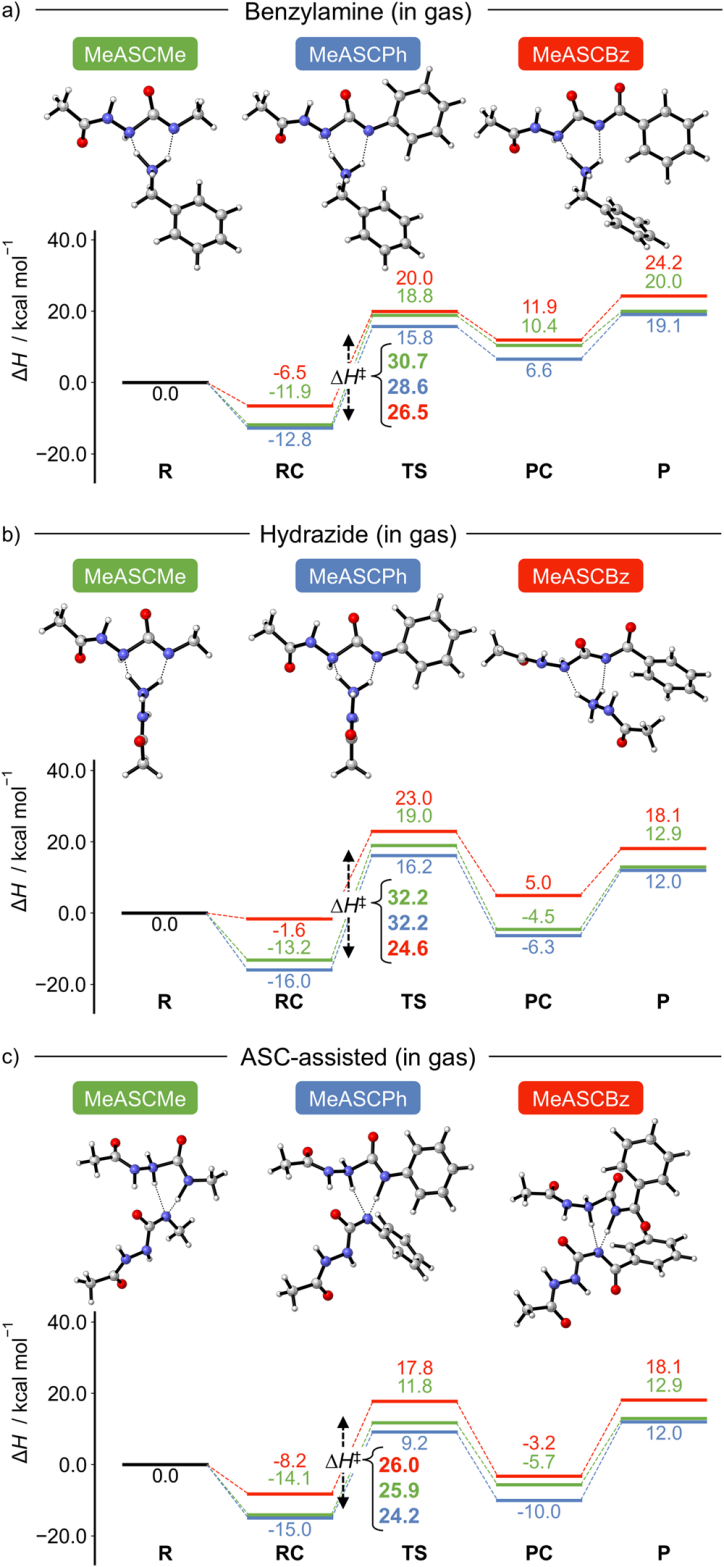
Potential energy surfaces (Δ*H*, in kcal mol^−1^) of the (a) benzylamine‐, (b) hydrazide‐, and (c) dimer‐assisted dissociation of MeASCMe, MeASCPh, and MeASCBz compounds, including their transition state structures. Dissociation always occurs on the ASC compound on top. Computed at ZORA‐M06‐2X/TZ2P//ZORA‐BLYP‐D3(BJ)/TZ2P in gas phase.

### 2.6 Opportunities for Tuning Polymer‐network Dynamics Enabled by Improved Mechanistic Understanding

Experimental model studies in combination with computational studies are an established and powerful tool to understand the intricacies of dynamic covalent bond exchange processes [8, 21, 22, 23, 24]. Such integrated studies enable the elucidation of reaction pathways and the identification of key intermediates [5, 8, 21, 23, 25] or reveal the assistance of functional groups or small molecules that facilitate the exchange process [22, 24, 26, 27]. Moreover, these studies aid in assessing how subtle changes in molecular structure impact the dynamic behavior at a material level [8, 10, 24]. Our model studies indicate that the presence of water or other proton‐shuttling species accelerates bond exchange between **ASC**s. Consequently, proton shuttling species are likely to affect dynamic exchange processes in polymer networks containing **ASC**s as well.

In a polymer material, however, it is difficult to experimentally discern the contribution of such effects, as many factors play a role in network dynamics, such as secondary interactions, network topology, and impurities [28, 29]. By comparing literature examples employing **ASC**s in different network environments, we find hints that proton shuttling effects may indeed play a role at the network level. Zuilhof and Xia synthesized **ASC**‐based polyethylene/propylene oxide (PEO/PPO) networks with a characteristic relaxation time τ (a parameter that measures how quickly material stress relaxes via bond exchange) around 60 s at 140°C [11]. Our previous studies showed τ values at 140°C around 750 and 1500 s in different types of **ASC**‐based polydimethylsiloxane (PDMS) networks [30, 31].

Although this difference of more than an order of magnitude likely arises from multiple contributing factors, there are distinct features in each system that will influence proton shuttling. First, in the polar PEO/PPO networks, water can more easily diffuse through the network structure than in the apolar PDMS‐based networks, weakening hydrogen bonds and speeding up the dynamic covalent relaxation. Second, the polarity of the PEO/PPO backbone likely results in a looser packing of **ASC** groups than in the strongly phase‐separated PDMS‐based networks, allowing hydrazide‐ or dimer‐assisted dissociation. Both factors will result in an increase in the bond exchange and relaxation rate in the PEO/PPO networks compared to the PDMS networks. These insights can be translated to other dynamic covalent systems bearing proton‐shuttling effects or other dynamic reaction‐assisting mechanisms and help improve our abilities to rationally design networks and control their relative relaxation rates.

## 3 Conclusion

In our previous work considering the dissociation of acylsemicarbazides (R^1^─CO─NHNH···CONH─R^2^) [10], it was identified that the R^2^ substantially influences the dissociation rate, whereas the influence of the R^1^ was much less pronounced. Through systematically changing the R^1^ and R^2^ substituents and combining experimental and computational methods, we show in this work that the influence of substituent variation at the R^2^ position can be rationalized by considering the stabilization of the partial negative charge that forms during proton transfer. Crucially, a causal relation is established between the proton affinities and the stabilization of the negative charge; substituents with stronger electron‐withdrawing character stabilize this negative charge more effectively, which leads to significantly higher dissociation rates. Given that proton affinity is a widely employed and intuitive metric, especially with its closeness to acidity and pK_a_ values, this correlation offers a straightforward tool for predicting experimental trends without the need for extensive computations. Consequently, our analysis of proton affinities and R^2^ substituent effects provides an easily transferable framework for guiding the design and optimization of other dynamic covalent bonds.

Moreover, we show in this work that the experimental dissociation rate of **ASC** cannot be explained considering the previously‐postulated unimolecular mechanism [11], regardless of solvent effects. Instead, species capable of mediating proton transfer, including water and various amine‐containing compounds, can facilitate the key step in **ASC** dissociation by accepting the proton from the isocyanate group and donating a proton to the hydrazide group within the same process. This mediation lowers the activation barrier to values that match the experimental rates, which has not been recognized previously for **ASC** motifs. In polymeric materials and dynamic covalent networks based on **ASC**s, neighboring **ASC** motifs as well as the hydrazide dissociation products can similarly act as internal proton‑transfer catalysts. Taken together, the mechanistic understanding developed here extends to a wide range of dynamic covalent systems with proton‑shuttling or related reaction‑assisting mechanisms, facilitating the rational design of networks and control of their relaxation behavior.

## Conflicts of Interest

The authors declare no conflicts of interest.

## Supporting information




**Supporting File 1**: chem71403‐sup‐0001‐SuppMat.pdf

**Supporting File 2**: chem71403‐sup‐0002‐DataFile.zip

## Data Availability

The data that supports the findings of this study are available in the supplementary material of this article.
