# Supplementary material for: Theoretical Framework for Predicting Dynamic Covalent Exchange Based on Proton Affinities and Proton‐Transfer Assistance
Source: Chemistry. 2026 Jul 22;32(40):e71403. doi: 10.1002/chem.71403 (PMC13715997; doi:10.1002/chem.71403)
Supplement: Supplementary file 1 [file CHEM-32-e71403-s002.pdf]

# Supporting Information for

# Theoretical framework for predicting dynamic covalent exchange based on proton affinities and proton-transfer assistance

Siebe Lekanne Deprez+,[a] Stefan J. D. Maessen+,[b] Angelina N. van Dam,[a] Celine Nieuwland,[a] Pascal Vermeeren,[a] Anja R. A. Palmans,*[b] Célia Fonseca Guerra*[a]

[a] Department of Chemistry and Pharmaceutical Sciences, AIMMS, Vrije Universiteit Amsterdam, De Boelelaan 1108, Amsterdam 1081 HZ, The Netherlands. E-mail: c.fonsecaguerra@vu.nl.

[b] Department of Chemical Engineering & Chemistry, and Institute for Complex Molecular Systems, Eindhoven University of Technology, P.O. Box 513, 5600 MB Eindhoven, The Netherlands. E-mail: a.palmans@tue.nl.

## Table of Contents


**S1 Theoretical Methods** **3**

S1.1 Computational Details 3

S1.2 Calculation of the potential energy surface (PES) 3

S1.3 Bond Energy Analysis 4

S1.4 Decomposition of the electrostatic interaction term $\Delta V_{electrostatics}$ 5

S1.5 Reaction rate derivation (first-order kinetics) 6

S1.6 Relationship between activation energy and computed barriers 7

**S2 Supporting Figures and Tables** **9**

S2.1 Proton affinity correlates values 9

S2.2 Estimated enthalpy barriers 9

S2.3 Proton affinity energy decomposition analysis 10

S2.4 Additional PES profiles 13

S2.5 Additional barrier of DMSO assistance 16

**S3 Materials and Methods** **18**

S3.1 Materials 18

S3.2 NMR solvent preparation 18

S3.3 Karl-Fischer titration 18

S3.4 NMR spectroscopy 18

S3.5 In-situ NMR kinetics 19

S3.6 Synthetic procedures 21

**S4 NMR and MALDI-ToF-MS** **22**

**S5 Cartesian coordinates** **27**

**S6 References** **27**

## S1 Theoretical Methods

### S1.1 Computational Details

All calculations were carried out using the Amsterdam Density Functional (ADF) 2025.103 module of the Amsterdam Modeling Suite.[1–4] All stationary points and energies were calculated at the BLYP level of the generalized gradient approximation (GGA) exchange functional developed by Becke (B[5]), and the GGA correlation functional developed by Lee, Yang, and Parr (LYP[6]). The DFT-D3(BJ) method developed by Grimme and co-workers[7], which contains the damping function proposed by Becke and Johnson[8], is used to describe non-local dispersion interactions. In addition, relativistic effects are accounted for through the zeroth-order regular approximation (ZORA) method.[9,10] This level of theory is referred to as ZORA-BLYP-D3(BJ)/TZ2P and has been proven to accurately describe weak interactions.[11–14] A large uncontracted relativistically-optimized TZ2P basis set was used with no frozen-core approximation consisting of Slater type orbitals (STOs) which is of triple-ζ quality for all atoms and has been augmented with the following sets of polarization functions: *p* and *d* functions on H, *d* and *f* functions on N, C, O.[15] The molecular density is fitted by the systematically improvable Zlm fitting scheme and numerical integration is performed on a Becke grid.[16,17] Both are specified with the "Good" option.

Conformational analyses of the $R^1$ASC$R^2$ monomers were performed using CREST[18] at a temperature of 100°C. Conformers with an energy of +2.0 kcal mol$^{-1}$ relative to the lowest one were neglected and duplicates were removed based on their RSMD (<0.05). The conformers were optimized at the above DFT level in gas phase and in DMSO using the implicit solvation model COSMO[19–22] and default atom radii (Allinger's atom radii divided by a factor of 1.2)[23].

To confirm that the stationary points are in their lowest energy conformation, frequency analyses have been carried out with zero imaginary frequencies for equilibrium geometries.[24–26] Transition-state points were confirmed to have one imaginary frequency with their vibrational modes corresponding to the desired reaction coordinate. Thermochemical corrections were calculated for the enthalpy *H*, entropy *S*, and Gibbs free energy *G* using the default ADF implementation. Finally, the geometries have been visualized using the CYLview20 program.[27]

### S1.2 Calculation of the potential energy surface (PES)

We define the following energies computed at a ZORA-BLYP-D3(BJ)/TZ2P level of theory:

$$E := E^{\text{BLYP-D3(BJ)}} \tag{S1}$$

$$H := H^{\text{BLYP-D3(BJ)}} \tag{S2}$$

$$G := G^{\text{BLYP-D3(BJ)}} \tag{S3}$$

Consequently, electronic energies were calculated on a higher level of theory being ZORA-M06-2X/TZ2P with the M06-2X hybrid XC-functional, with 54% Hartree-Fock exchange. The M06-2X

XC-functional performs better than BLYP-D3(BJ) for kinetic studies in which bonds are broken and formed, which occurs during the proton transfers we study in this article.[28] But, due to the high computation costs of calculating (numerical) frequencies with M06-2X, no frequencies and thermodynamic corrections were calculated. Instead, the thermodynamic corrections to *H* and *G* obtained at ZORA-BLYP-D3(BJ)/TZ2P were added to the electronic energies obtained at ZORA-M06-2X/TZ2P, resulting in the label ZORA-M06-2X/TZ2P//ZORA-BLYP-D3(BJ)/TZ2P in which the part after the // denotes at which level the geometry is optimized.

$$E^{\text{M06-2x}} = E^{\text{M06-2X}} \tag{S4}$$

$$H^{\text{M06-2x}} = E^{\text{M06-2X}} + (H - E) \tag{S5}$$

$$G^{\text{M06-2x}} = E^{\text{M06-2X}} + (G - E) \tag{S6}$$

Consequently, the relative energies, and hence the PES profile, were computed by subtracting the sum of all reactant energies from the energy of each stationary point denoted by X:

$$\Delta E^{\text{X}} = E^{\text{X}} - \sum E^{\text{R}} \text{ with X = R, RC, INT, PC, P} \tag{S7}$$

We define the energy barrier as the energy difference between the transition state, and the adjacent reactant (complex, R/RC) and/or intermediate (INT) to which it is connected.

$$\Delta H^{\ddagger} = H^{\text{M06-2X, TS}} - H^{\text{M06-2X, RC/INT/PC}} \tag{S8}$$

We consider enthalpies instead of Gibbs free energies since the former is directly related to the experimental dissociation rates through the Eyring-Polanyi equation[29] (see section S1.6).

### S1.3 Bond Energy Analysis

The bond energy, $\Delta E$, can be partitioned according to the activation strain model (ASM) formulated by eqn. S9:[30–34]

$$\Delta E = \Delta E_{\text{strain}} + \Delta E_{\text{interaction}} \tag{S9}$$

Here, the strain energy ($\Delta E_{\text{strain}}$) represents the energetic cost of deforming each fragment from its equilibrium geometry to the geometry adopted in the complex. The interaction energy ($\Delta E_{\text{interaction}}$) accounts for stabilizing interactions between deformed fragments. We can decompose the interaction energy further within the framework of the Kohn-Sham molecular orbital model (KS-MO), using the quantitative canonical energy decomposition analysis (EDA[35,36]). We identify the electrostatic interaction ($\Delta V_{\text{electrostatics}}$), Pauli repulsion ($\Delta E_{\text{Pauli}}$), orbital interactions

($\Delta E_{\text{orbital interactions}}$), and an additional term that accounts for dispersion interactions $\Delta E_{\text{dispersion}}$, as shown in eqn. S10.

$$\Delta E_{\text{int}} = \Delta V_{\text{electrostatics}} + \Delta E_{\text{Pauli}} + \Delta E_{\text{orbital interactions}} + \Delta E_{\text{dispersion}} \tag{S10}$$

The $\Delta V_{\text{electrostatics}}$ term denotes the quasi-classical Coulomb interaction between the unperturbed charge distributions of the deformed fragments and it usually attractive (*i.e.*, a negative contribution) for neutral fragments. $\Delta E_{\text{Pauli}}$ comprises destabilizing interactions between occupied orbitals on each fragment and is responsible for steric repulsion (*i.e.*, a positive contribution). The orbital interaction energy ($\Delta E_{\text{orbital interactions}}$) accounts for donor-acceptor interactions and polarization effects between and within the fragments, including interactions between the highest occupied and lowest unoccupied MOs (HOMO–LUMO). These interactions cause the electrons to "relax" and stabilize, hence being attractive. Finally, $\Delta E_{\text{dispersion}}$ accounts for dispersion corrections as introduced by Grimme *et al.*[7,8] This decomposition provides a quantitative framework for understanding the relative contributions of electrostatics, steric effects, orbital interactions, and dispersion forces to molecular interactions.

### S1.4 Decomposition of the electrostatic interaction term $\Delta V_{\text{electrostatics}}$

The electrostatic interaction, $\Delta V_{\text{electrostatics}}$, between two molecular fragments A and B can be further decomposed into four contributions: nuclei–nuclei ($\Delta V_{\text{N-N}}$), nuclei–electrons ($\Delta V_{\text{N-e}}$), electrons–nuclei ($\Delta V_{\text{e-N}}$), and electron–electron ($\Delta V_{\text{e-e}}$) electrostatic interaction:

$$\Delta V_{\text{electrostatics}} = \Delta V_{\text{N-N}} + \Delta V_{\text{N-e}} + \Delta V_{\text{e-N}} + \Delta V_{\text{e-e}}$$

$$\Delta V_{\text{electrostatics}} = \sum_{\alpha \in A} \sum_{\beta \in B} \frac{Z_\alpha Z_\beta}{R_{\alpha\beta}} - \int \sum_{\alpha \in A} \frac{Z_\alpha \rho^B(r)}{|R_\alpha - r|} dr - \int \sum_{\beta \in B} \frac{Z_\beta \rho^A(r)}{|R_\beta - r|} dr + \int\int \frac{\rho^A(r_1)\rho^B(2)}{r_{12}}\, dr_1 dr_2 \tag{S11}$$

The first term, $\Delta V_{\text{N-N}}$, represents the interaction between the nuclei belonging to fragment A and fragment B. This term is always positive as the nuclei repel each other with like charges. The second term, $\Delta V_{\text{N-e}}$, indicates the attractive interaction between nuclei belonging to fragment A and the electron density of fragment B. The third term, $\Delta V_{\text{e-N}}$ is similar to $\Delta V_{\text{N-e}}$ but with the fragments interchanged. The last term, $\Delta V_{\text{e-e}}$, represents the repulsion between the two electron densities of fragment A and B, respectively.

### S1.5 Reaction rate derivation (first-order kinetics)

Earlier studies indicate first-order kinetics for the dissociation of **ASC** motifs.[37,38] Deriving an expression for the observed rate is achieved through the following steps. First, we write the general form associated with first-order kinetics:

$$k_d \cdot [A] = -\frac{d[A]}{dt} \text{(first order kinetics)}$$

With $k_d$ the dissociation rate, and $[A]$ the concentration of species $A$ at a given time $t$. Grouping terms together by multiplying both sides by $dt$ and dividing by $[A]$ gives:

$$k_d \cdot dt = -\frac{d[A]}{[A]}$$

Integrating both sides and evaluating the integrals yields:

$$k_d \cdot \int dt = -\int \frac{d[A]}{[A]}$$
$$k_d \cdot (t - t_0) = -(\ln[A] - \ln[A]_0)$$

We choose $t_0$ = 0, and applying the logarithmic division rule:

$$k_d t = -\ln\left(\frac{[A]}{[A]_0}\right)$$

Finally, we are interested in determining the rate from experimental data in which we track the concentration (with respect to the starting concentration) for a time period. Taking the negative slope of the curve yields the dissociation rate:

$$k_d = -\frac{\Delta \ln\left(\frac{[A]}{[A]_0}\right)}{\Delta \mathrm{t}} \quad \text{(slope in discretized form)} \qquad (\mathrm{S12})$$

In conclusion, the slope of $\ln\left(\frac{[A]}{[A]_0}\right)$ with respect to time yields the expression for the **ASC** dissociation rate at a constant temperature. This relation has been applied to calculate the rates at various temperatures.

## S1.6 Relationship between activation energy and computed barriers

The aim of this section is to derive a relation between experimental dissociation rates and computable quantities such as the Gibbs free energy of activation, $\Delta G^{\ddagger}$, enthalpy of activation, $\Delta H^{\ddagger}$, and entropy of activation, $\Delta S^{\ddagger}$. First, in the framework of transition state theory (TST[39]), the experimental dissociation rates, $k_d$, can be related to the equilibrium constant, $K^{\ddagger}$, through

$$k_d = \frac{\kappa k_B T}{h} K^{\ddagger} \qquad \text{(S13)}$$

with $k_d$ denoting the measured dissociation rate, $\kappa$ the transmission coefficient (unitless), $k_b$ Boltzmann's constant (in J·K$^{-1}$), $T$ the temperature (in K), and $h$ Planck's constant (in J·s). To reach an expression in terms of the $\Delta G^{\ddagger}$, $\Delta H^{\ddagger}$, or $\Delta S^{\ddagger}$, we consider the relation between the Gibbs free energy and the equilibrium constant:

$$\Delta G^{\ddagger} = -RTln\left(K^{\ddagger}\right) \rightarrow K^{\ddagger} = e^{-\frac{\Delta G^{\ddagger}}{RT}} \qquad \text{(S14)}$$

where $R$ is the gas constant (in J·mol$^{-1}$·K$^{-1}$). Note that we refer to standard thermodynamic quantities, $\Delta X^{\ddagger\ominus}$, but we omit the state sign to simplify notation. Substituting eqn. S13 into S14 yields the Eyring-Polanyi equation:[29]

$$k_d = \frac{\kappa k_B T}{h} e^{-\frac{\Delta G^{\ddagger}}{RT}} \qquad \text{(S15)}$$

We proceed by substituting $\Delta G^{\ddagger} = \Delta H^{\ddagger} - \mathrm{T}\Delta S^{\ddagger}$,

$$k_d = \frac{\kappa k_B T}{h} e^{-\frac{\Delta H^{\ddagger}}{RT}} e^{\frac{\Delta S^{\ddagger}}{R}} \qquad \text{(S16)}$$

dividing both sides by $T$, and taking the natural logarithm:

$$\ln\left(\frac{k_d}{T}\right) = \underbrace{\frac{-\boldsymbol{\Delta H^{\ddagger}}}{\boldsymbol{RT}}}_{\text{Slope}} \cdot \frac{1}{T} + \underbrace{\ln\left(\frac{\kappa k_b}{h}\right) + \frac{\Delta S^{\ddagger}}{R}}_{\text{y-intercept}} \qquad \text{(S17)}$$

Eqn. S17 is a promising expression because the temperatures and rates can be measured experimentally, and the enthalpy of activation $\Delta H^{\ddagger}$ can be computed. One can even estimate $\Delta H^{\ddagger}$ from rates determined at different temperatures: if $\ln\left(\frac{k_d}{T}\right)$ is plotted against $\frac{1}{T}$, the slope of the

(linear) curve is related to the enthalpy of activation by $\frac{-\Delta H^{\ddagger}}{RT}$. Thus, we have established that the experimental rates can be related to the enthalpy barrier $\Delta H^{\ddagger}$ through the Eyring-Polanyi equation.

## S2 Supporting Figures and Tables

### S2.1 Proton affinity correlates values

**Table S1.** Relative experimental reaction rates ($k_d$) and proton affinity (PA) calculations (in kcal mol$^{-1}$) of **R$^1$ASCR$^2$** monomers (R$^1$ = Me, Ph; R$^2$ = Me, Ph, Bz) in the gas phase. The rates and relative energies, $\Delta\Delta E$, are calculated with respect to **MeASCMe** and **PhASCMe**. Enthalpy values are computed at ZORA-BLYP-D3(BJ)/TZ2P.

| System | $k_d$ | $-\Delta H$ (=PA) | $-\Delta\Delta H$ (=ΔPA) |
|---|---|---|---|
| **MeASCMe** | 1.0 | 181.0 | 0.0 |
| **MeASCPh** | 17.4 | 175.3 | –5.7 |
| **MeASCBz** | 215.5 | 167.3 | –13.7 |
| | | | |
| **PhASCMe** | 1.0 | 178.3 | –2.6 |
| **PhASCPh** | 20.8 | 173.5 | –7.5 |
| **PhASCBz** | 219.3 | 166.8 | –14.1 |

### S2.2 Estimated enthalpy barriers

**Table S2.** Calculation of estimated enthalpy of activation $\Delta H^{\ddagger}$ for the dissociation of **R$^1$ASCR$^2$** compounds with R$^1$ = Me, and Ph, and R$^2$ = Me, Ph, and Bz, from experimental dissociation rates at different temperatures (in K), see eqn. S17.

| | | **MeASCMe** | **MeASCPh** | **MeASCBz** |
|---|---|---|---|---|
| T | 1000/T | ln($k_d$/T) | ln($k_d$/T) | ln($k_d$/T) |
| 373.15 | 2.68 | -19.2362 | -16.3771 | -13.8630 |
| 383.15 | 2.61 | -18.3032 | -15.5627 | -15.0406 |
| 393.15 | 2.54 | -17.4405 | -14.6832 | -16.1082 |
| 403.15 | 2.48 | -16.4936 | -13.7613 | -17.4109 |
| $-\Delta H^{\ddagger}$ / RT [J mol$^{-1}$] | | –13.67 | –13.11 | –15.00 |
| $\Delta H^{\ddagger}$ [kcal mol$^{-1}$] | | 27.2 | 26.1 | 29.8 |
| | | **PhASCMe** | **PhASCPh** | **PhASCBz** |
| T | 1000/T | ln($k_d$/T) | ln($k_d$/T) | ln($k_d$/T) |
| 373.15 | 2.68 | -19.0296 | -15.9943 | -13.6389 |
| 383.15 | 2.61 | -18.0813 | -14.9958 | -14.6695 |
| 393.15 | 2.54 | -17.2324 | -14.1719 | -15.8289 |
| 403.15 | 2.48 | -16.4430 | -13.1918 | -16.9137 |
| $-\Delta H^{\ddagger}$ / RT [J mol$^{-1}$] | | –12.96 | –13.88 | –14.06 |
| $\Delta H^{\ddagger}$ [kcal mol$^{-1}$] | | 25.7 | 27.6 | 27.9 |

### S2.3 Proton affinity energy decomposition analysis

**Table S3.** Activation strain model analyses (ASM) and energy decomposition analyses (EDA) of **R¹ASCR²** proton affinity calculations ($R^1$ = Me, Ph; $R^2$ = Me, Ph, Bz). Energy values are reported in kcal mol$^{-1}$ and the N–H distance, $r_{N–H}$, is reported in Å, computed at ZORA-BLYP-D3(BJ)/TZ2P.

| | | ASM | | | EDA | | | |
|---|---|---|---|---|---|---|---|---|
| System | $r_{N–H}$ | $\Delta E$ | $\Delta E_{strain}$ | $\Delta E_{interaction}$ | $\Delta V_{electrostatics}$ | $\Delta E_{Pauli}$ | $\Delta E_{orbital\ interactions}$ | $\Delta E_{disp}$ |
| **MeASCMe** | 1.0141 | –355.0 | 11.4 | –366.4 | –162.7 | 0.0 | –202.7 | –1.0 |
| **MeASCPh** | 1.0149 | –342.4 | 9.5 | –351.9 | –144.5 | 0.0 | –206.1 | –1.3 |
| **MeASCBz** | 1.0148 | –334.1 | 12.4 | –346.5 | –138.6 | 0.0 | –206.5 | –1.5 |
| | | | | | | | | |
| **PhASCMe** | 1.0141 | –352.2 | 11.0 | –363.3 | –153.4 | 0.0 | –208.9 | –1.0 |
| **PhASCPh** | 1.0149 | –340.6 | 9.5 | –350.1 | –139.7 | 0.0 | –209.2 | –1.3 |
| **PhASCBz** | 1.0148 | –332.9 | 12.5 | –345.4 | –135.2 | 0.0 | –208.7 | –1.5 |

We can further decompose the electrostatic interaction, $\Delta V_{electrostatics}$, between the **R¹ASCR²** anion and proton ($H^+$) into four terms: the repulsive terms $\Delta E_{nuc\text{-}nuc}$ and $\Delta E_{elec\text{-}elec}$ , and the attractive terms $\Delta E_{elec\text{-}nuc}$ and $\Delta E_{nuc\text{-}elec}$ (see Section S1.4). Note that, for these systems, $\Delta E_{elec\text{-}elec}$ and $E_{nuc\text{-}elec}$ do not contribute as the $H^+$ does not contain any electrons. The remaining terms, $\Delta E_{nuc\text{-}nuc}$ and $\Delta E_{elec\text{-}nuc}$, represent the nuclear repulsion between the nuclei of the anion and the proton, and the attraction between the density on the anion with the proton, respectively. Table S4 shows the numerical values and we observe that $\Delta E_{nuc\text{-}nuc}$ becomes more destabilizing (*i.e.*, more positive) and $\Delta E_{elec\text{-}nuc}$, becomes more stabilizing from $R^2$ = Me, Ph, and Bz. To establish the dominating term, we computed the ratios of the two terms with respect to the $\Delta E_{nuc\text{-}nuc}$ term for obtaining a fixed reference and calculated the difference $\Delta\Delta E_{nuc\text{-}nuc}$ = (ratio of $\Delta E_{nuc\text{-}nuc}$) – (ratio of $\Delta E_{nuc\text{-}nuc}$) to observe how the terms change compared to the reference (see Table S4). The procedure was carried out **MeASCR²** and **PhASCR²** separately with $R^2$ = Me, Ph, and Bz.

The results show that the nuclear repulsion increasing more rapidly than the electron-nuclei attraction: **MeASCPh** has a $\Delta E_{nuc\text{-}nuc}$ term that is 0.337 more repulsive than for **MeASCMe**, but also a $\Delta E_{elec\text{-}nuc}$ term that is 0.335 times more attractive. Similarly, for **MeASCBz**, $\Delta E_{nuc\text{-}nuc}$ is 0.510 times more repulsive and $\Delta E_{elec\text{-}nuc}$ 0.507 times more attractive than for **MeASCMe.** The same trend is observed for the **PhASCR²** series with $R^2$ = Me, Ph, Bz. Thus, the stronger electrostatic interactions upon varying the $R^2$ substituent from Me to Ph, to Bz stem from a faster increasing of the nuclear repulsion between the nuclei of the anion and proton compared to the attraction between additional electrons on the anion and the proton.

**Table S4.** Decomposition of the electrostatic interaction term[a] $\Delta V_{electrostatics}$ of **R[1]ASCR[2]** ($R^1$ = Me, Ph; $R^2$ = Me, Ph, Bz). Energy values are reported in kcal mol$^{-1}$, computed at ZORA-BLYP-D3(BJ)/TZ2P using "Excellent" BeckeGrid and STOFit instead of ZlmFit.

| System | $\Delta E_{nuc\text{-}nuc}$ | $\Delta E_{elec\text{-}nuc}$ | ratio $\Delta E_{nuc\text{-}nuc}$[a] | ratio $\Delta E_{elec\text{-}nuc}$[a] | $\Delta\Delta E_{nuc\text{-}nuc}$[b] | $\Delta\Delta E_{elec\text{-}nuc}$[b] |
|---|---|---|---|---|---|---|
| **MeASCMe** | 8669 | –8834 | 1.000 | –1.019 | | |
| **MeASCPh** | 11591 | –11733 | 1.337 | –1.353 | 0.337 | –0.335 |
| **MeASCBz** | 13093 | –13223 | 1.510 | –1.526 | 0.510 | –0.507 |
| | | | | | | |
| **PhASCMe** | 10017 | –10170 | 1.000 | –1.015 | | |
| **PhASCPh** | 12952 | –13089 | 1.293 | –1.307 | 0.293 | –0.291 |
| **PhASCBz** | 14456 | –14589 | 1.443 | –1.457 | 0.443 | –0.441 |

[b] Ratios of $\Delta E_{nuc\text{-}nuc}$ and $\Delta E_{elec\text{-}nuc}$ are computed with respect to solely $\Delta E_{nuc\text{-}nuc}$ relative to **R[1]ASCMe** with $R^1$ = Me and Ph.
For example: ratio of $\Delta E_{nuc\text{-}nuc}^{MeASCPh} = \Delta E_{nuc\text{-}nuc}^{MeASCPh} / \Delta E_{nuc\text{-}nuc}^{MeASCMe}$.
[c] $\Delta\Delta E_{nuc\text{-}nuc}$ and $\Delta\Delta E_{elec\text{-}nuc}$ are computed with respect to the ratios of $\Delta E_{nuc\text{-}nuc}$ and $\Delta E_{elec\text{-}nuc}$ relative to **R[1]ASCMe** with $R^1$ = Me and Ph.
For example: $\Delta\Delta E_{nuc\text{-}nuc}^{MeASCPh}$ = ratio of $\Delta E_{nuc\text{-}nuc}^{MeASCPh}$ – ratio of $\Delta E_{nuc\text{-}nuc}^{MeASCMe}$

The reason why nuclear-nuclear repulsion increases faster than the electron-nuclear stabilization in the order $R^2$ = Me, Ph, Bz, may be because $\pi$-conjugation with anionic **R[1]ASC** moiety increases, withdrawing more electron density as a result. This would cause the electron-nuclear stabilization to stabilize slower as electron density interacts less significantly with the nucleus of $H^+$. To support this statement, we consider the molecular electrostatic potential map (MEP) of the anionic, deprotonated species (see Figure S1). Since the $H^+$ is considered a point charge and contains no electrons, the MEP is directly related to $\Delta V_{electrostatics}$ and its decomposition terms.

Figure S1 shows the results for $R^1ASCR^2$ with $R^1$ = Me, Ph, and Bz, and $R^2$ = Me and Ph. Indeed, the MEP values correlate well with the trend in $\Delta V_{electrostatics}$ since both becomes less negative when varying $R^2$ (from left to right in Figure S1). Moreover, the MEP shows both numerically and visually that the electrostatic potential near the deprotonated N atom (in the direction and with the length of the cleaved N–H bond) is most stabilizing for **MeASCMe** (–161.1 kcal mol$^{-1}$) and least stabilizing for **PhASCBz** (–135.2 kcal mol$^{-1}$) since the MEP near the deprotonated N atom is the most red for **MeASCMe** while for **PhASCBz** the least red. Thus, the MEPs support the hypothesis that the phenyl and benzoyl substituents withdraw more electrons as their MEP values near the deprotonated N atom align with the trend in $\Delta V_{electrostatics}$, with benzoyl exerting the biggest influence.

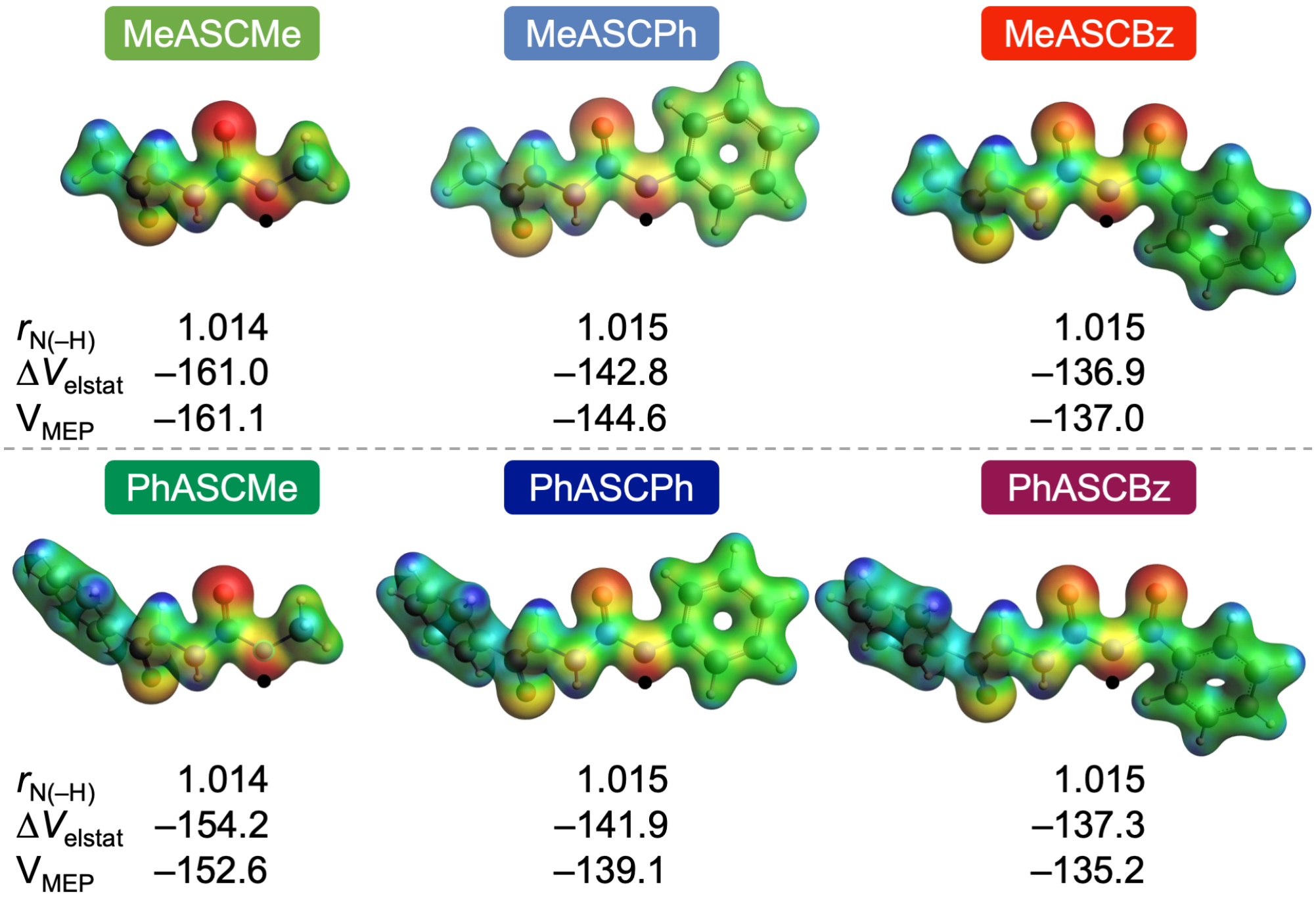


**Figure S1.** Molecular electrostatic potential maps (MEPs) of **R[1]ASCR[2]** anions made by projecting the molecular electrostatic potential $V_{MEP}$ onto an isosurface of the density. The isovalues of the densities were chosen such that the isosurface intersects the $r_{N–H}$ distance in the complex (in Å), indicated by the black dots. The corresponding $V_{MEP}$ values range from –125.5 kcal mol$^{-1}$ (red) to +125.5 kcal mol$^{-1}$ (blue). Computed at ZORA-BLYP-D3(BJ)/TZ2P.

## S2.4 Additional PES profiles

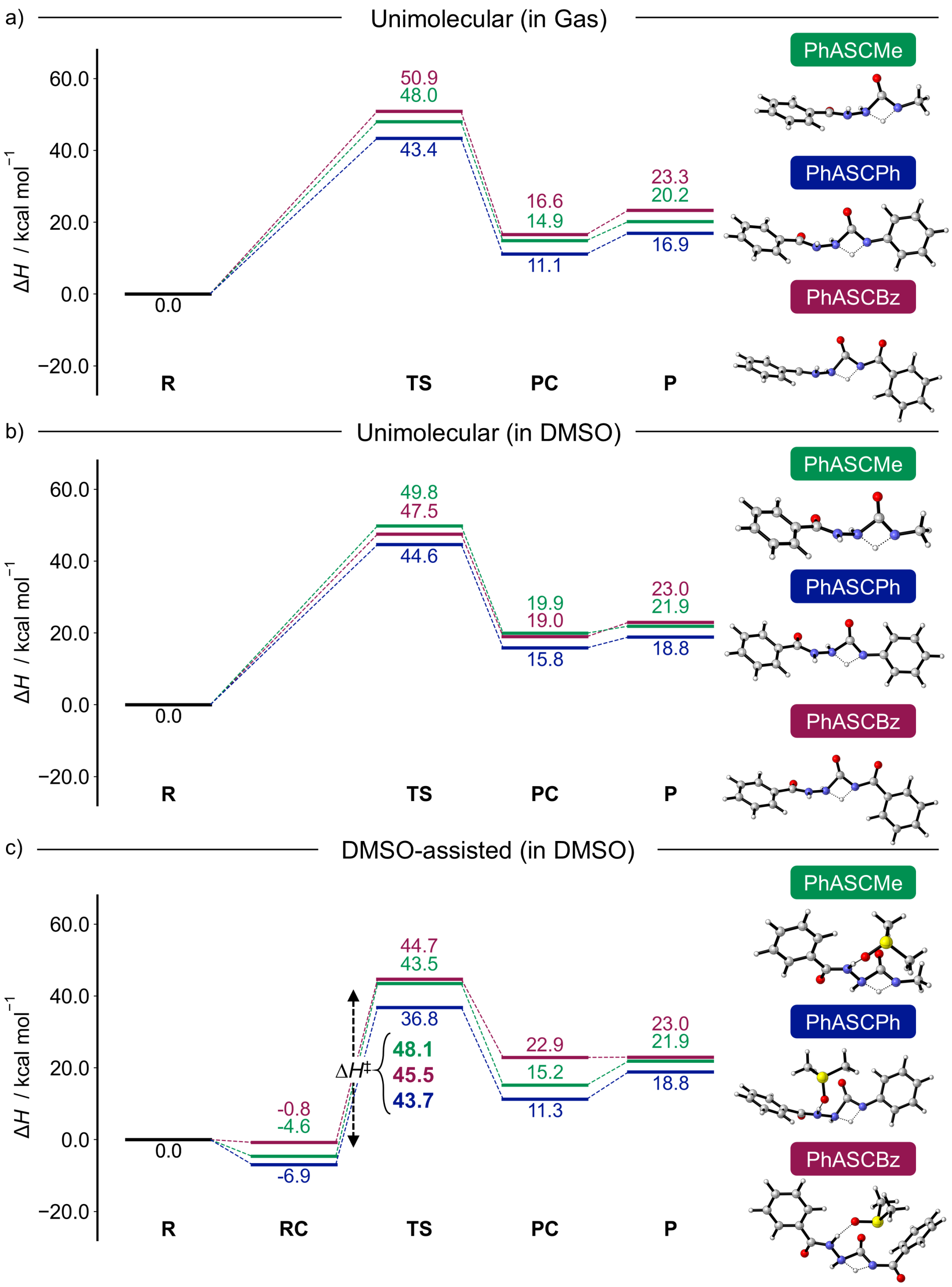


**Figure S2**. Potential energy surfaces ($\Delta H$, in kcal mol$^{-1}$) computed for the unimolecular dissociation of **PhASCR$^2$** monomers in a) the gas phase, b) DMSO, and c) explicit DMSO. Transition-state structures are shown next to the PES, computed at [COSMO(DMSO)]-ZORA-M06-2X/TZ2P//[COSMO(DMSO)]-ZORA-BLYP-D3(BJ)/TZ2P.

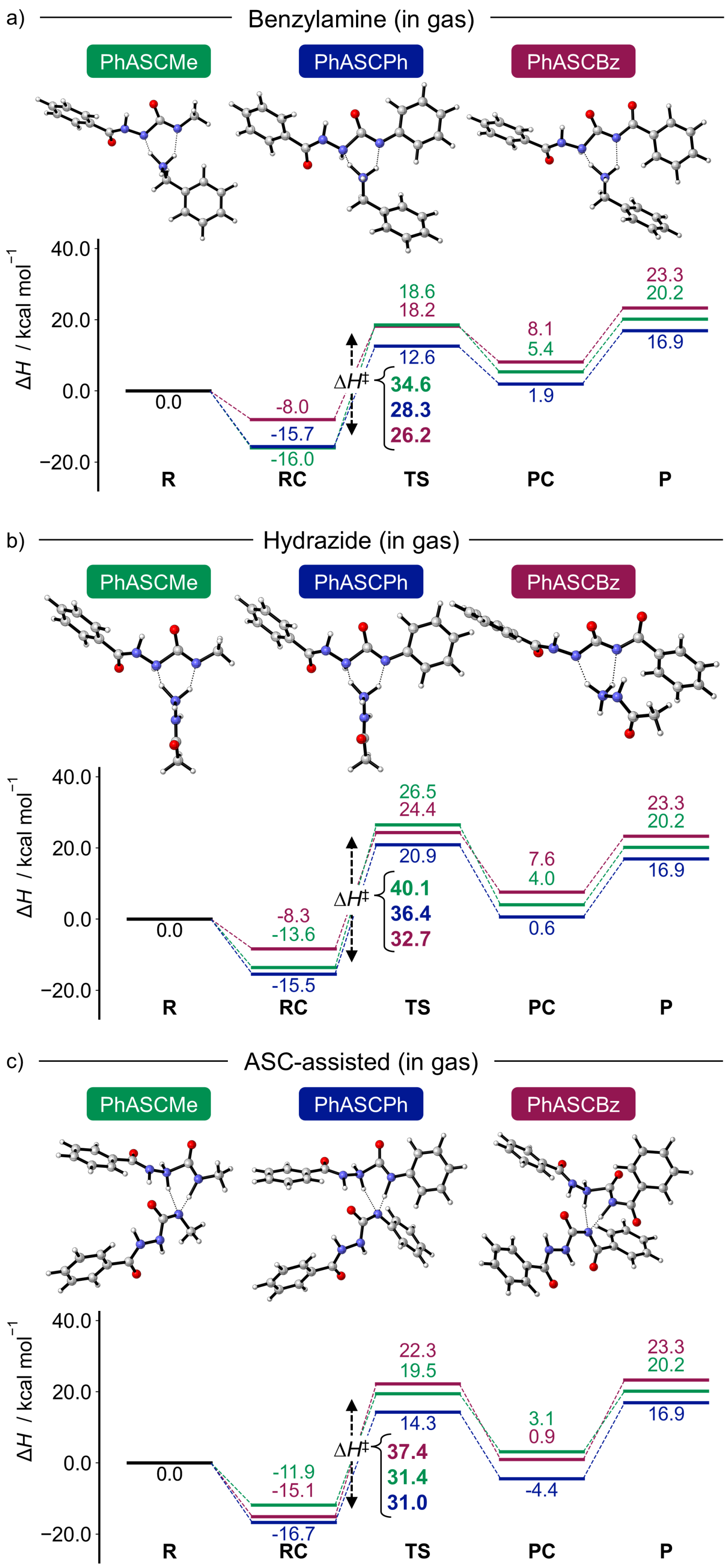


**Figure S3.** Potential energy surfaces (Δ*H*, in kcal mol$^{-1}$) of the a) benzylamine-, b) hydrazide-, and c) dimer-assisted dissociation of **PhASCMe**, **PhASCPh**, and **PhASCBz** compounds, including their transition state structures. Dissociation always occurs on the ASC compound on top. Computed at ZORA-M06-2X/TZ2P//ZORA-BLYP-D3(BJ)/TZ2P in gas phase.

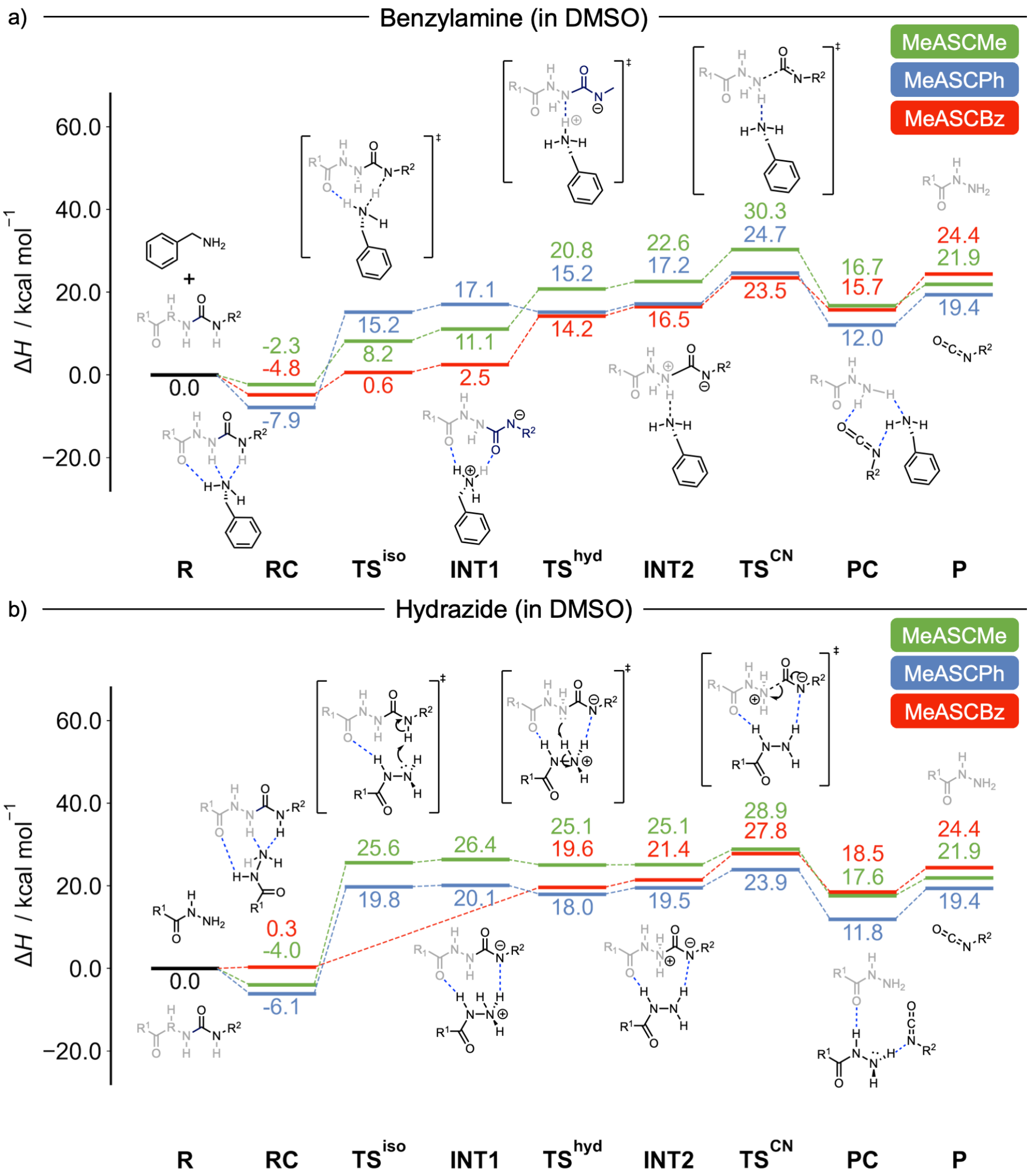


**Figure S4.** Potential energy surfaces (Δ*H*, in kcal mol$^{-1}$) of the a) benzylamine- and b) hydrazide-assisted dissociation of **MeASCMe**, **MeASCPh**, and **MeASCBz** compounds, computed at COSMO(DMSO)-ZORA-M06-2X/TZ2P//COSMO(DMSO)-ZORA-BLYP-D3(BJ)/TZ2P.

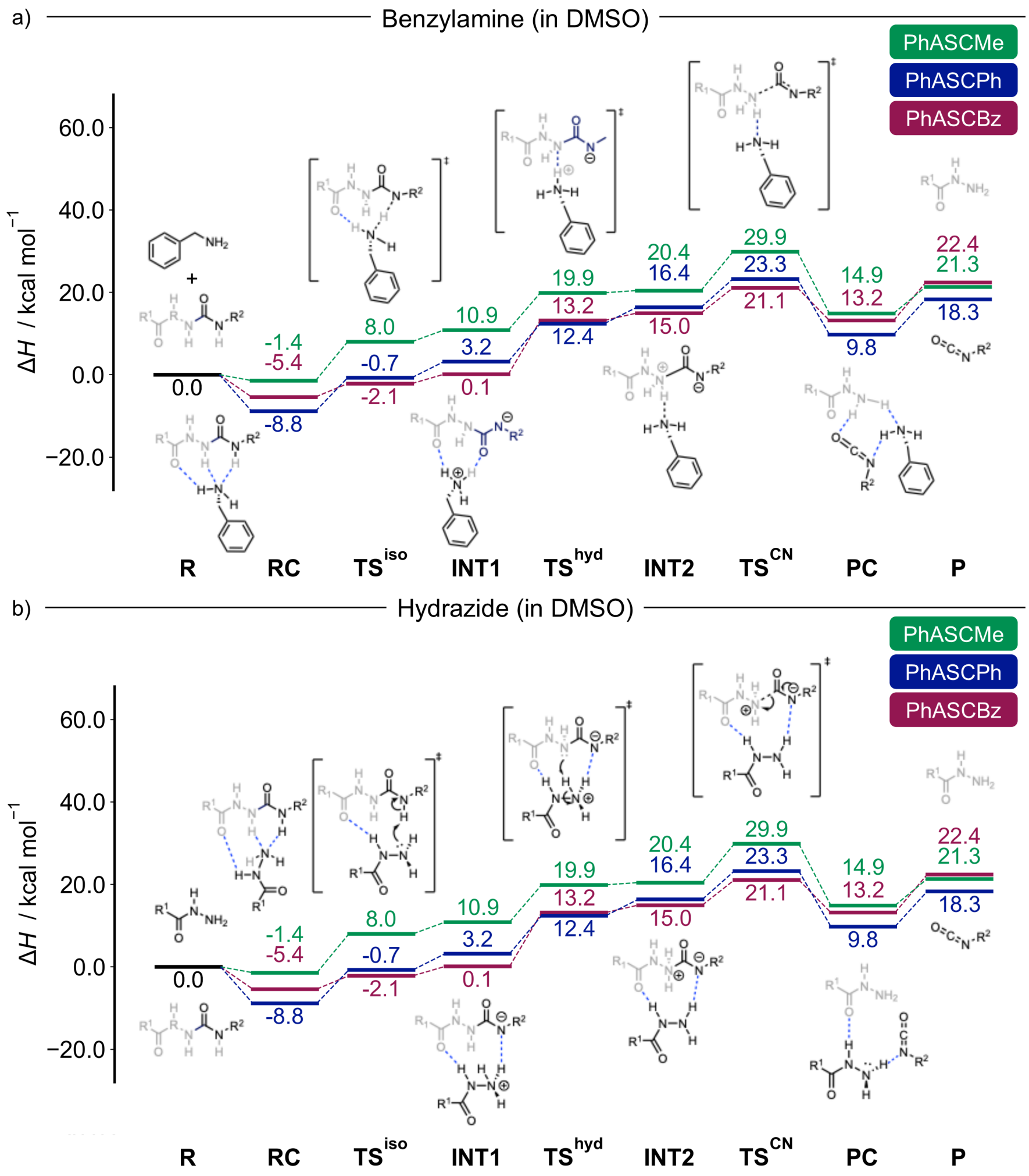


**Figure S5.** Potential energy surfaces ($\Delta H$, in kcal mol$^{-1}$) of the a) benzylamine- and b) hydrazide-assisted dissociation of **PhASCMe**, **PhASCPh**, and **PhASCBz** compounds, computed at COSMO(DMSO)-ZORA-M06-2X/TZ2P//COSMO(DMSO)-ZORA-BLYP-D3(BJ)/TZ2P.

## S2.5 Additional barrier of DMSO assistance

We report in the main article that DMSO does not assist the dissociation because i) the enthalpy barriers are too high and ii) the "assisting" DMSO molecule does not bind directly to the active site (see Figure 3 in the main article and Figure S3). We highlight the associated transition-state structures that show a DMSO molecule forming an hydrogen bond with the N–H group of the **HN**-NH-C(=O) part of the **ASC** monomer (see also Figure S6a and Figure S7a). One may wonder if DMSO can lower the dissociation barrier by coordinating to the other N–H bond, HN-**NH**-C(=O). However, we find that the dissociation barriers increase. Through performing transition-state searches, valid transition-state structures were obtained (*i.e.*, with one imaginary frequency) that are shown in Figure S6b and Figure S7b. The enthalpy barrier, $\Delta H^{\ddagger}$, is higher for the hydrogen bond between HN-**NH**-C(=O) and DMSO than for the hydrogen bond between **HN**-NH-C(=O) and DMSO. This occurs for both the **MeASCR$^2$** (Figure S6) and **PhASCR$^2$** series (Figure S7) with $R^2$ = Methyl, Phenyl, and Benzoyl. Thus, the enthalpy barrier is not lowered, but raised instead if a

DMSO molecule is coordinated closer to the active site, *i.e.*, where the proton transfer occurs. Thus, we conclude that DMSO does not assist in the **ASC** dissociation.

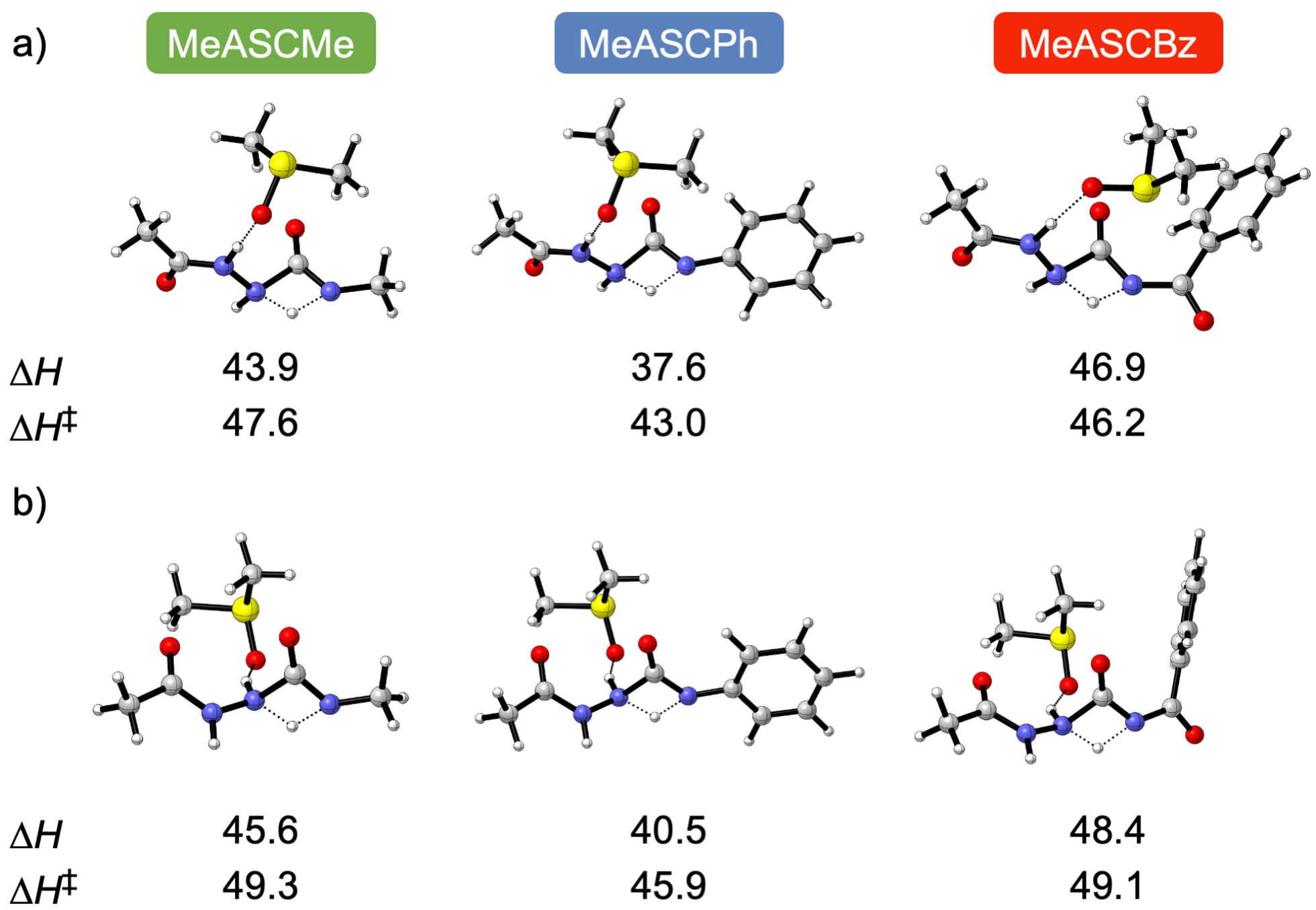


**Figure S6.** Transition-state structures of DMSO-assisted dissociation of **MeASCR[1]** with $R^1$ = Methyl, Phenyl, and benzoyl including enthalpies $\Delta H$ (in kcal mol$^{-1}$), and enthalpy barriers, $\Delta H^‡$ (in kcal mol$^{-1}$) relative to the reactant complex. a) The lowest barrier and b) a higher-laying barrier, computed at COSMO(DMSO)-ZORA-M06-2X/TZ2P//COSMO(DMSO)-ZORA-BLYP-D3(BJ)/TZ2P.

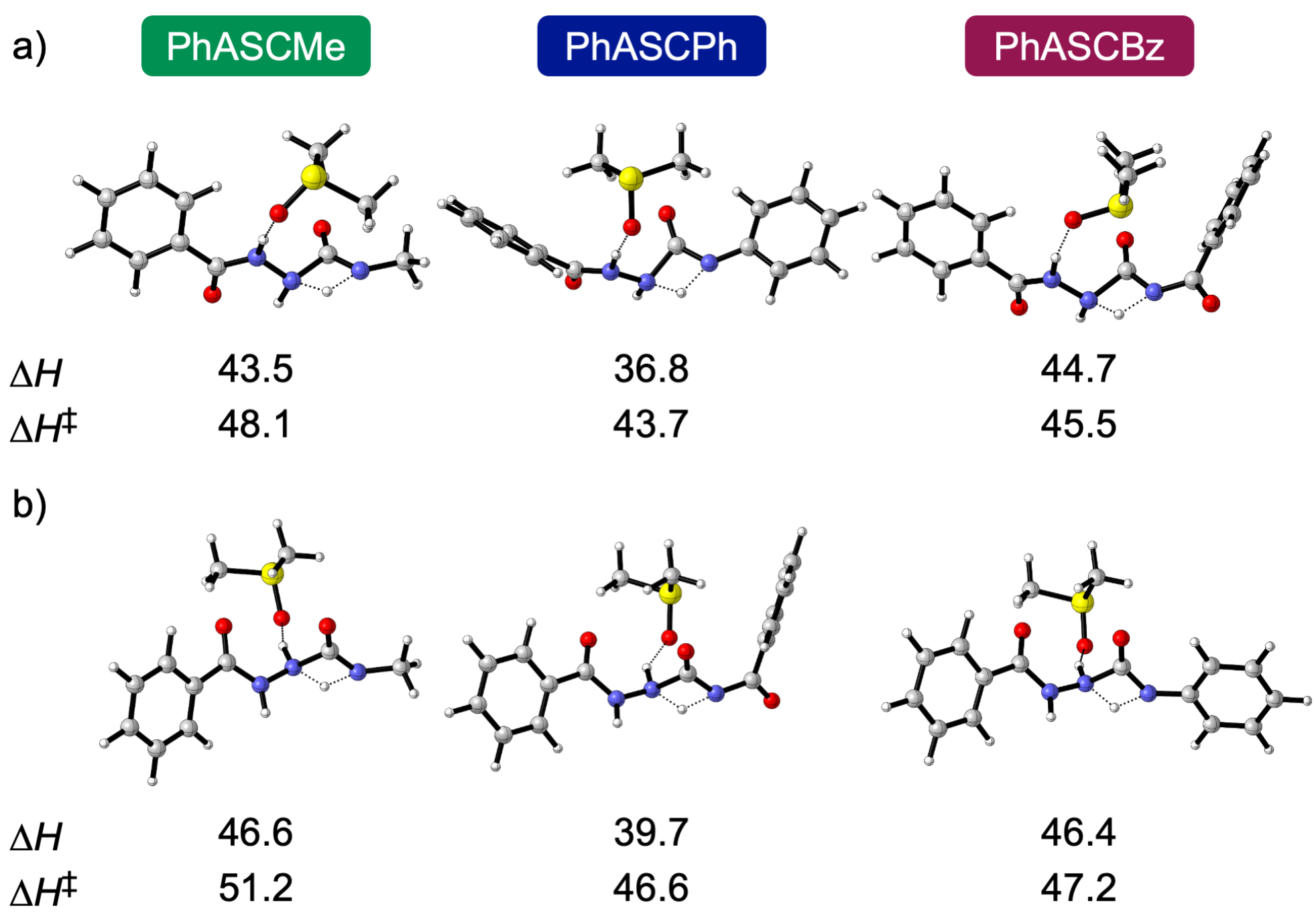


**Figure S7.** Transition-state structures of DMSO-assisted dissociation of **PhASCR[1]** with $R^1$ = Methyl, Phenyl, and benzoyl including enthalpies $\Delta H$ (in kcal mol$^{-1}$), and enthalpy barriers, $\Delta H^‡$ (in kcal mol$^{-1}$) relative to the reactant complex. a) The lowest barrier and b) a higher-laying barrier, computed at COSMO(DMSO)-ZORA-M06-2X/TZ2P//COSMO(DMSO)-ZORA-BLYP-D3(BJ)/TZ2P.

## S3 Materials and Methods

### S3.1 Materials

All reagents and chemicals were obtained from commercial sources at the highest purity available and used without further purification. All solvents were obtained from Biosolve except for acetonitrile which was obtained from Actu-All Chemicals. Deuterated DMSO (DMSO-$d_6$) and deuterated TCE (TCE-$d_2$) were purchased from Deutero GmbH and used as received. Anhydrous DMSO-$d_6$ (<50 ppm) was purchased from Sigma Aldrich and further dried on activated 3 Å molsieves in a glovebox.

### S3.2 NMR solvent preparation

**Wet DMSO-$d_6$** was prepared by using a regularly used bottle of DMSO-$d_6$ without any special storage.

**Dry DMSO-$d_6$** was prepared by further drying anhydrous DMSO-$d_6$ (<50 ppm) on activated 3 Å molsieves in a glovebox.

**Wet TCE-$d_2$** was prepared by mixing 10 mL of TCE-$d_2$ with 10 mL of demineralized water, and vigorously shaking and sonicating the mixture for 15 minutes. After the layers had separated, the wet TCE-$d_2$ was separated from the water via syringe and used as is.

**Dry TCE-$d_2$** was prepared by further drying TCE-$d_2$ on activated 4 Å molsieves in a glovebox.

### S3.3 Karl-Fischer titration

The water content present in the NMR solvents used in this study were measured using a Mettler-Toledo C30 Coulometric KF Titrator loaded with CombiCoulomat Frit KF reagent (for cells with diaphragm, contains ethanol, purchased from Merck).

For each measurement, a syringe was loaded with approximately 0.5 mL of solvent. The syringe was weighed, and the solvent was injected into the Karl-Fischer titrator. The emptied syringe was weighed again to calculate the amount of injected solvent. Each measurement was replicated three times, and the obtained values were averaged to give the following water contents: Wet DMSO-$d_6$ (1070 ppm), Dry DMSO-$d_6$ (36 ppm), Wet TCE-$d_2$ (990 ppm) and dry TCE-$d_2$ (19 ppm).

### S3.4 NMR spectroscopy

$^1$H, $^{13}$C NMR spectra were recorded on a 400 MHz Bruker Avance III Spectrometer ($^1$H using 400 MHz, $^{13}$C using 100 MHz). Peak multiplicities are abbreviated as s: singlet, d: doublet, t: triplet, q: quartet. Proton and carbon chemical shifts are reported in parts per million (ppm) downfield from

tetramethylsilane (TMS) using the deuterated solvent resonance frequency as internal standard (2.50 ppm for DMSO-$d_6$).

### S3.5 In-situ NMR kinetics

In-situ $^1$H NMR spectra at elevated temperatures were recorded on a Varian Unity Inova 500 MHz Spectrometer. The NMR probe was pre-heated before loading the samples, and sample were equilibrated for at least 5 minutes before starting the measurement.

Dissociation kinetics of **ASC** model compounds were measured by making a stock solution of benzylamine in DMSO-$d_6$ or TCE-$d_2$. This stock solution was added to the **ASC** compounds to an **ASC** concentration of 25 mM. Subsequently, 0.4 mL of the mixture was transferred to an NMR tube and transferred to the NMR spectrometer. All samples containing dry solvents were prepared in a glovebox and measured using sealed Wilmad® quick pressure valve NMR tubes.

Dissociation rates were determined by following the decrease in intensity of an ASC model compound peak over time. The peaks used for integration is mentioned below the respective model compound in Table S5. Exceptions to this are **MeASCMe** and **PhASCMe**, where no clear disappearing peak was present. In these cases, denoted by a * in Table S5, the conversion was determined by the integral of the appearing $CH_3$-NH-CO-NH-<u>$CH_2$</u>-Ph proton signals. The integral of these peaks at 100% conversion point was determined using the integral of the **$R^1$ASC<u>Me</u>** peak with $R^1$ = Me or Ph.

As the system follows pseudo first-order kinetics, $k_d$ was determined from the slope of $\ln\left(\frac{c}{c_0}\right)$ as a function of time (see section S1.5, eqn. S12). The activation energy, $E_{act}$, was determined from the slope of $\ln(k_d)$ as a function of inverse temperature $\frac{1}{T}$ (eqn. S18).

$$k_d = Ae^{-\frac{E_{act}}{RT}} \rightarrow \ln(k_d) = \ln(A) - \frac{\boldsymbol{E_{act}}}{\boldsymbol{R}}\frac{1}{T} \quad \text{(S18)}$$

The uncertainty in $E_{act}$, denoted $\sigma_{E_{act}}$, was determined by standard error propagation obtained from the linear regression, giving $\sigma_{E_{act}} = R \cdot \sigma_m$, where $\sigma_m$ is the standard error of the slope obtained from Origin's least-squares fit. Multiplication by the gas constant $R$ ensures consistent units with $E_{act}$.

**Table S5.** Model compound kinetics data. Shift of tracked peak indicated below the corresponding **ASC** model compound (in ppm).

| ASC compound | Solvent (-) | Wet/dry (-) | Eq. BA (-) | Temperature (°C) | $k_d$ (min$^{-1}$) | $E_{act}$ (kJ mol$^{-1}$) |
|---|---|---|---|---|---|---|
| **MeASCMe**<br>$\delta$ = 4.25* | DMSO-$d_6$ | Wet | 3 | 100 | $9.91*10^{-5}$ | 117 ± 3 |
| | | | | 110 | $2.59*10^{-4}$ | |
| | | | | 120 | $6.23*10^{-4}$ | |
| | | | | 130 | $1.66*10^{-3}$ | |
| **PhASCMe**<br>$\delta$ = 4.25* | DMSO-$d_6$ | Wet | 3 | 100 | $1.22*10^{-4}$ | 111 ± 1 |
| | | | | 110 | $3.23*10^{-4}$ | |
| | | | | 120 | $7.74*10^{-4}$ | |
| | | | | 130 | $1.75*10^{-3}$ | |
| **MeASCPh**<br>$\delta$ = 6.96 | DMSO-$d_6$ | Wet | 3 | 100 | $1.73*10^{-3}$ | 112 ± 4 |
| | | | | 110 | $4.01*10^{-3}$ | |
| | | | | 120 | $9.91*10^{-3}$ | |
| | | | | 130 | $2.55*10^{-2}$ | |
| **PhASCPh**<br>$\delta$ = 6.97 | DMSO-$d_6$ | Wet | 3 | 100 | $2.53*10^{-3}$ | 119 ± 3 |
| | | | | 110 | $7.06*10^{-3}$ | |
| | | | | 120 | $1.65*10^{-2}$ | |
| | | | | 130 | $4.51*10^{-2}$ | |
| **MeASCBz**<br>$\delta$ = 1.92 | DMSO-$d_6$ | Wet | 3 | 70 | $5.65*10^{-4}$ | 128 ± 3 |
| | | | | 80 | $2.14*10^{-3}$ | |
| | | | | 90 | $6.40*10^{-3}$ | |
| | | | | 100 | $2.14*10^{-2}$ | |
| **PhASCBz**<br>$\delta$ = 8.02 | DMSO-$d_6$ | Wet | 3 | 70 | $9.29*10^{-4}$ | 120 ± 2 |
| | | | | 80 | $2.83*10^{-3}$ | |
| | | | | 90 | $9.28*10^{-3}$ | |
| | | | | 100 | $2.67*10^{-2}$ | |
| **PhASCPh**<br>$\delta$ = 4.25* | TCE-$d_2$ | Wet | 3 | 110 | $1.73*10^{-2}$ | 106 ± 2 |
| | | | | 115 | $2.59*10^{-2}$ | |
| | | | | 120 | $3.84*10^{-2}$ | |
| | | | | 125 | $6.14*10^{-2}$ | |
| | | | | 130 | $8.81*10^{-2}$ | |
| $\delta$ = 6.97 in DMSO-$d_6$<br>**PhASCPh**<br>$\delta$ = 7.23 in TCE-$d_2$ | TCE-$d_2$ | Wet | 1 | 120 | $2.79*10^{-2}$ | |
| | | | 2 | 120 | $3.33*10^{-2}$ | |
| | | | 3 | 120 | $3.92*10^{-2}$ | |
| | | Dry | 1 | 120 | $2.16*10^{-2}$ | |
| | | | 2 | 120 | $3.21*10^{-2}$ | |
| | | | 3 | 120 | $3.72*10^{-2}$ | |
| | DMSO-$d_6$ | Wet | 1 | 120 | $1.55*10^{-2}$ | |
| | | | 2 | 120 | $1.67*10^{-2}$ | |
| | | | 3 | 120 | $1.73*10^{-2}$ | |
| | | Dry | 1 | 120 | $1.45*10^{-2}$ | |
| | | | 2 | 120 | $1.59*10^{-2}$ | |
| | | | 3 | 120 | $1.61*10^{-2}$ | |

### S3.6 Synthetic procedures

The syntheses and characterization of **MeASCPh**, **PhASCPh**, and **PhASCBz** have been reported in our previous work.[1]

### Synthesis of ASC model compounds

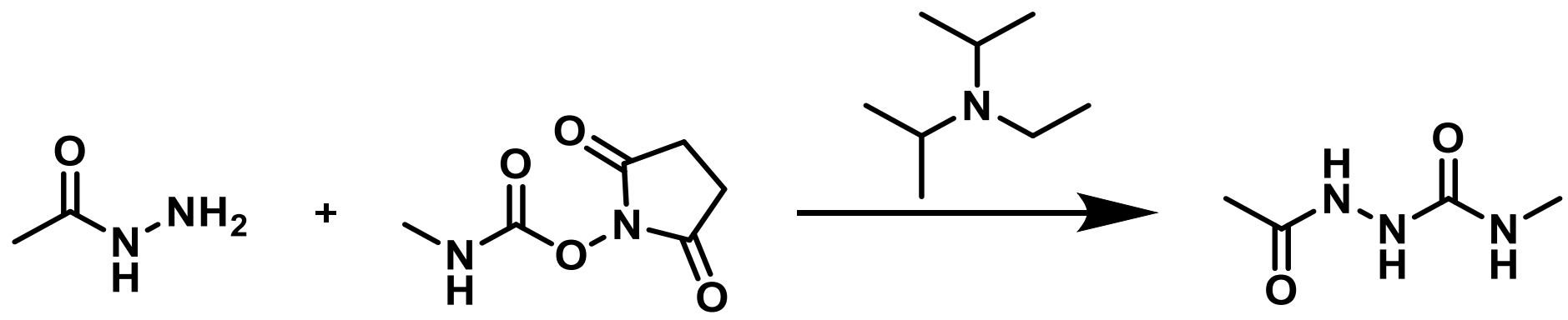

**MeASCMe:** Acethydrazide (1.0 g , 13.5 mmol, 1 eq) was added to a 50 mL round-bottom flask under argon. N-succimidyl N-methyl carbamate (2.56 g, 14.9 mmol, 1.8 eq), acetonitrile (ACN, 10 mL, dried on 3A molsieves) and N,N-diisopropylethylamine (DIPEA, 7.9 mL, 45.0 mmol, 3.0 eq) were added to the flask and the mixture was stirred at room temperature overnight. A white precipitate had formed, which was filtered using a Büchner filter and washed with 2x 10 mL of ACN and 2x 20 mL of diethyl ether. The product was dried in a vacuum oven, yielding a white solid (1.4 g, 79%). $^1$H NMR (400 MHz, DMSO-$d_6$) $\delta$ = 9.40 (s, 1H), 7.64 (s, 1H), 6.27 (d, $J$ = 4.9 Hz, 1H), 2.54 (d, $J$ = 4.6 Hz, 3H), 1.81 (s, 3H). $^{13}$C NMR (100 MHz, DMSO-$d_6$) $\delta$ = 169.66, 159.21, 26.63, 21.14. MALDI-ToF-MS: m/z calc. for $C_4H_9N_3O_2$: 131.07 Da; found 154.16 Da $[M+Na]^+$, 145.28 Da $[M-CH_2+Na]^+$.

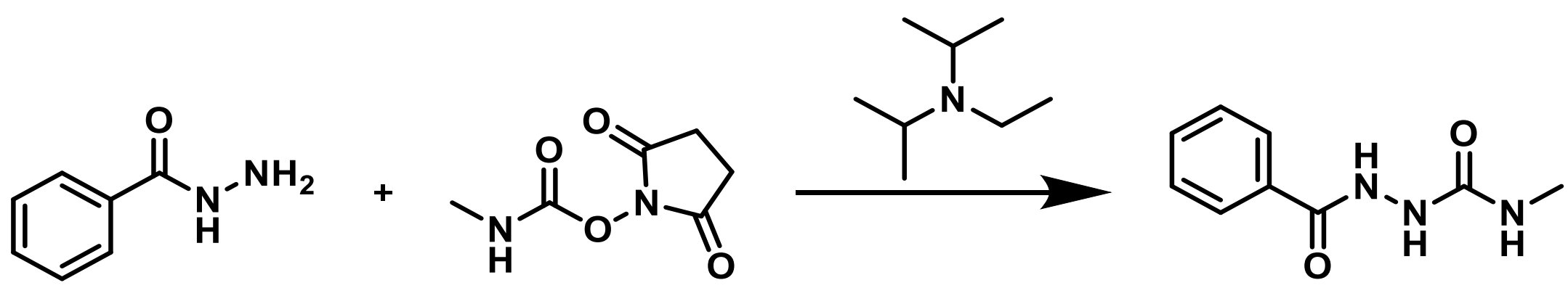

**PhASCMe:** Benzhydrazide (500 mg, 3.67 mmol, 1 eq) was added to a 50 mL round-bottom flask equipped with an oil bath and reflux cooler under argon. N-succimidyl N-methyl carbamate (1.16 g, 6.74 mmol, 1.8 eq), acetonitrile (ACN, 5 mL, dried on 3A molsieves) and N,N-diisopropylethylamine (DIPEA, 3.5 mL, 20.0 mmol, 5.5 eq) were added to the flask and the mixture was stirred at 50 °C overnight. The solvent was removed in *vacuo*, yielding a white powder which was suspended in 25 mL of DCM and filtered using a Büchner filter. The white powdszer was recrystallized from 8 mL of ACN, filtered using a Büchner filter, and washed with 2x 5 mL of ACN and 2x 5 mL of diethyl ether. The product was dried in a vacuum oven, yielding a white solid (160 mg, 23%). $^1$H NMR (400 MHz, DMSO-$d_6$) $\delta$ = 10.10 (s, 1H), 8.01 – 7.75 (m, 3H), 7.56 (t, $J$ = 7.3 Hz, 1H), 7.48 (t, $J$ = 7.5 Hz, 2H), 6.41 (q, $J$ = 4.6 Hz, 1H), 2.58 (d, $J$ = 4.5 Hz, 3H). $^{13}$C NMR (100 MHz, DMSO-$d_6$) $\delta$ = 166.84, 159.40, 133.23, 132.09, 128.73, 128.04, 26.73. MALDI-ToF-MS: m/z calc. for $C_9H_{11}N_3O_2$: 193.09 Da; found 194.16 Da $[M+H]^+$, 216.12 Da $[M+Na]^+$.

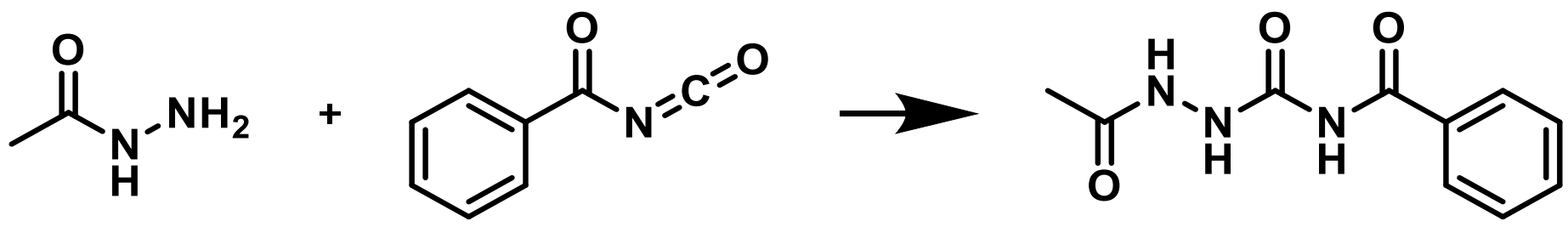

**MeASCBz:** Acethydrazide (200 mg, 2.70 mmol, 1 eq) was added to a 50 mL round-bottom under argon. Benzoyl isocyanate (397 mg, 2.70 mmol, 1 eq) and acetonitrile (ACN, 13 mL, dried on 3A molsieves) were added to the flask and the mixture was stirred at room temperature for 2 hours. A white precipitate had formed, which was filtered using a Büchner filter and washed with 2x 12 mL of ACN and 2x 12 mL of diethyl ether. The product was dried in a vacuum oven, yielding a white solid (511 mg, 86%). $^{1}$H NMR (400 MHz, DMSO-$d_6$) $\delta$ = 11.02 (s, 1H), 10.11 (s, 1H), 10.06 (s, 1H), 7.99 (d, $J$ = 7.1 Hz, 2H), 7.65 (t, $J$ = 7.4 Hz, 1H), 7.53 (t, $J$ = 7.7 Hz, 2H), 1.91 (s, 3H). $^{13}$C NMR (100 MHz, DMSO-$d_6$) $\delta$ = 168.47, 153.69, 133.45, 132.62, 129.03, 128.74, 20.84. MALDI-ToF-MS: m/z calc. for $C_{10}H_{11}N_3O_3$: 221.08 Da; found 222.13 Da $[M+H]^+$, 244.10 Da $[M+Na]^+$.

## S4 NMR and MALDI-ToF-MS

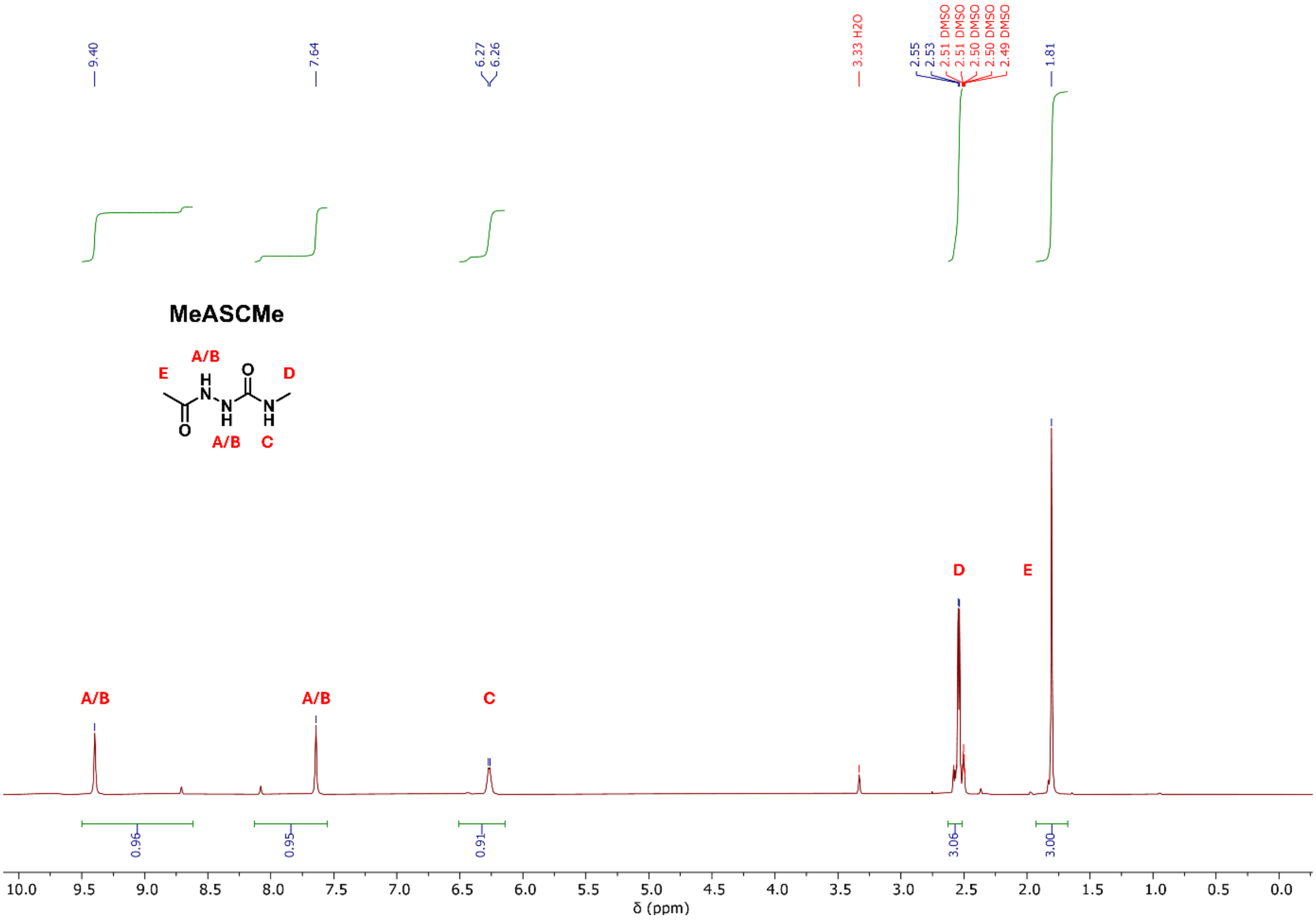


**Figure S8.** $^{1}$H NMR spectrum of **MeASCMe** in DMSO-$d_6$.

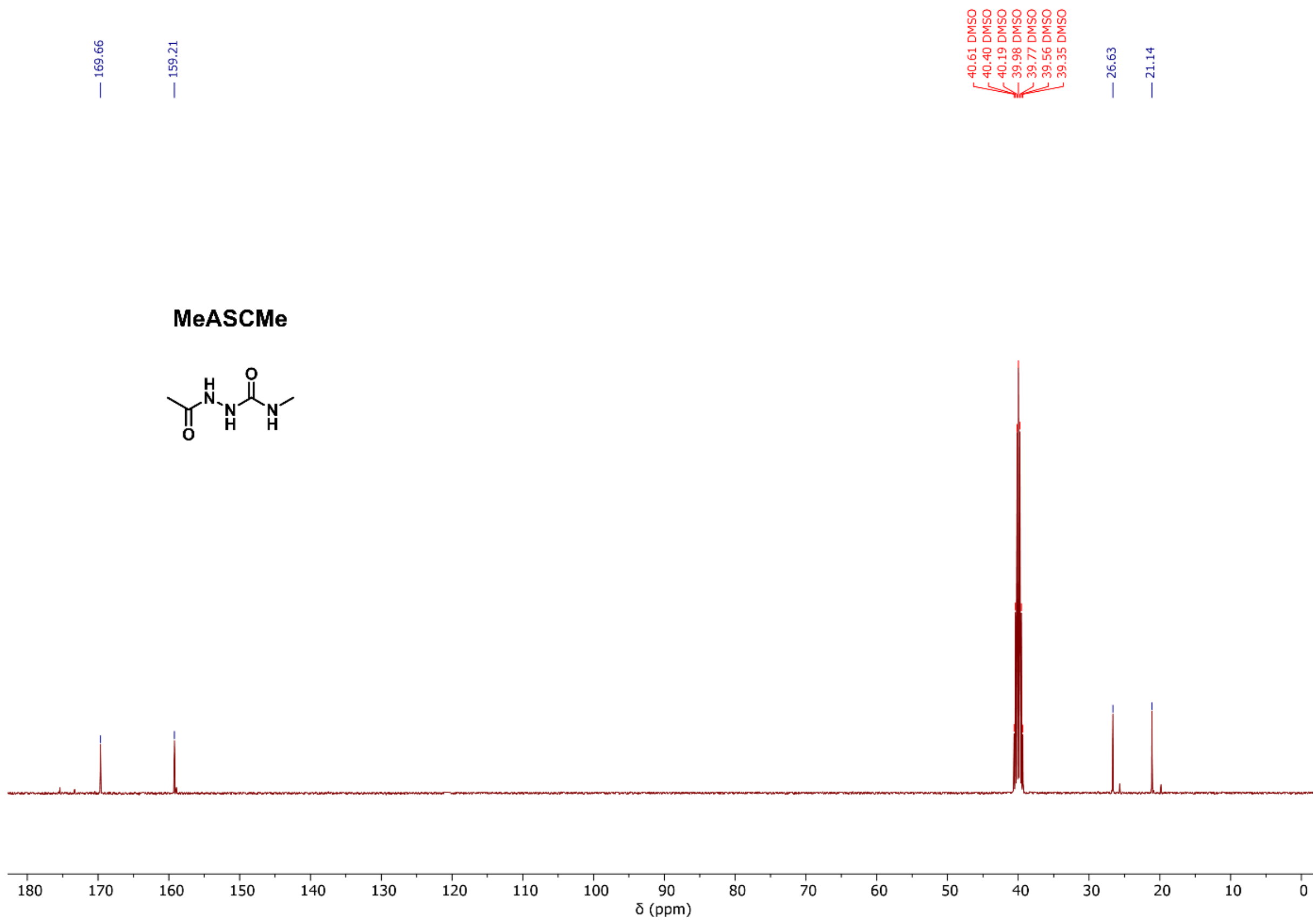


**Figure S9.** $^{13}$C NMR spectrum of **MeASCMe** in DMSO-$d_6$.

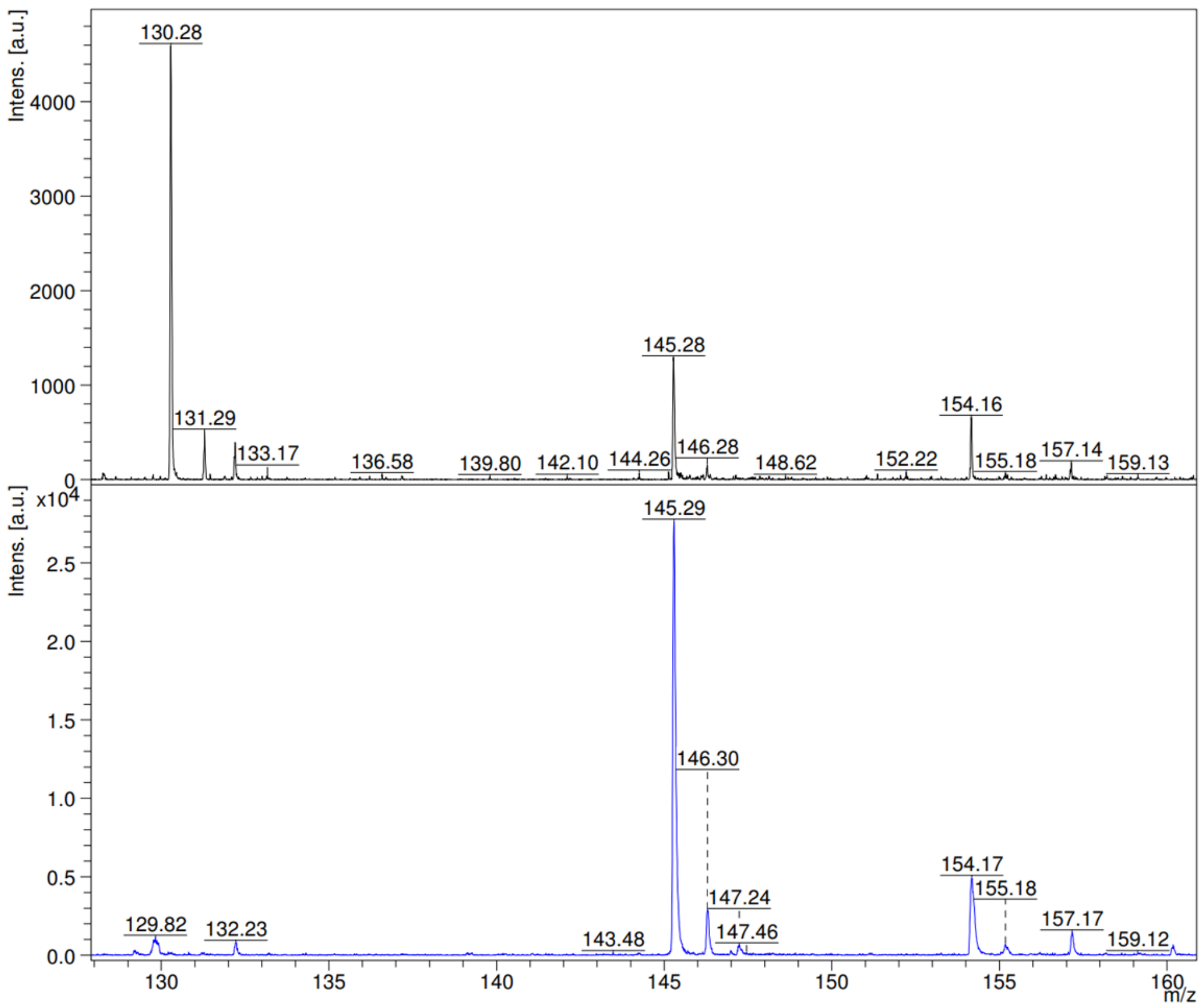


**Figure S10.** MALDI-ToF-MS spectra of **MeASCMe** (black: CHCA, blue: DCTB matrix).

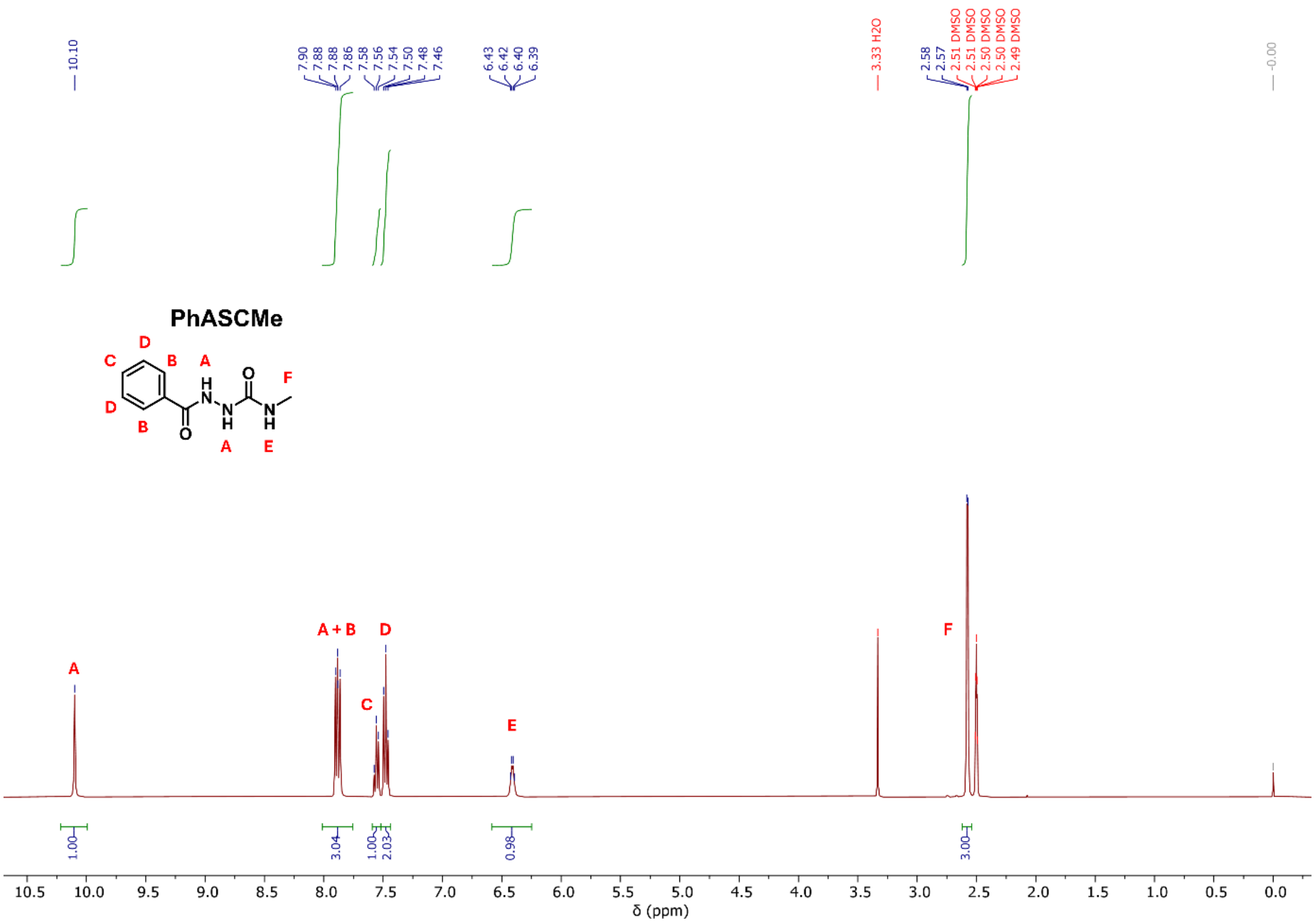

**Figure S11.** $^{1}H$ NMR spectrum of **PhASCMe** in DMSO-$d_6$.

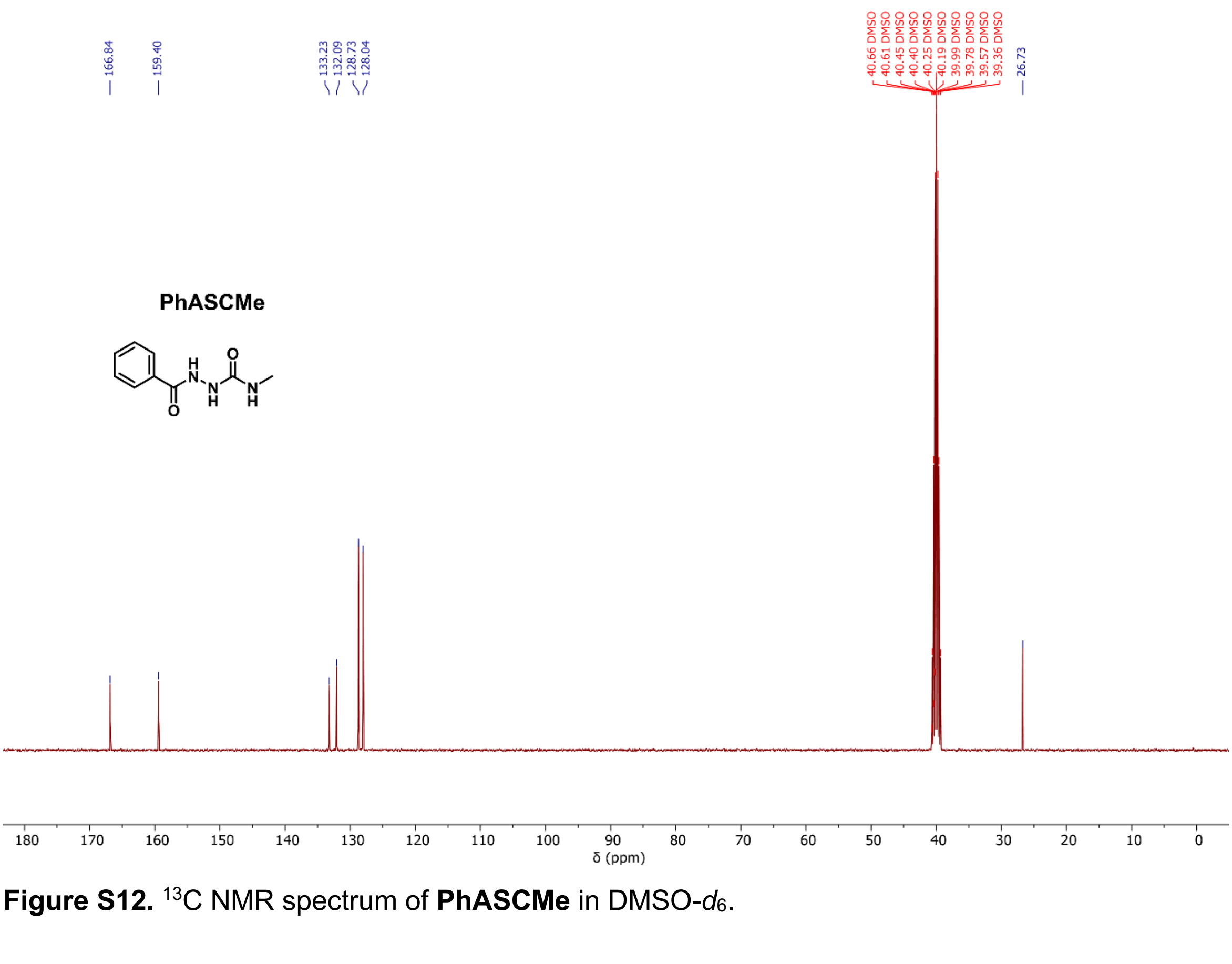

**Figure S12.** $^{13}C$ NMR spectrum of **PhASCMe** in DMSO-$d_6$.

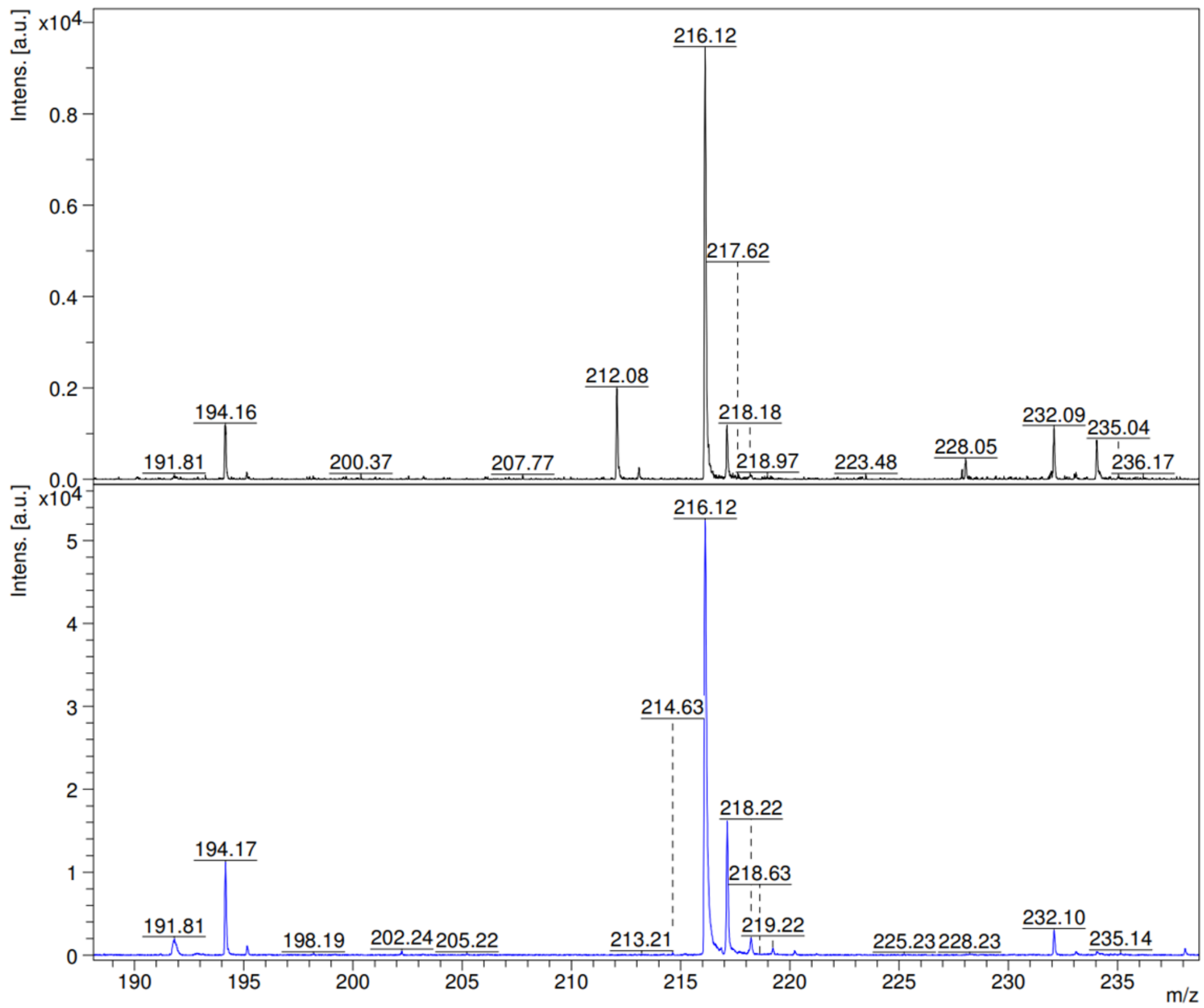


**Figure S13.** MALDI-ToF-MS spectra of **PhASCMe** (black: CHCA, blue: DCTB matrix).

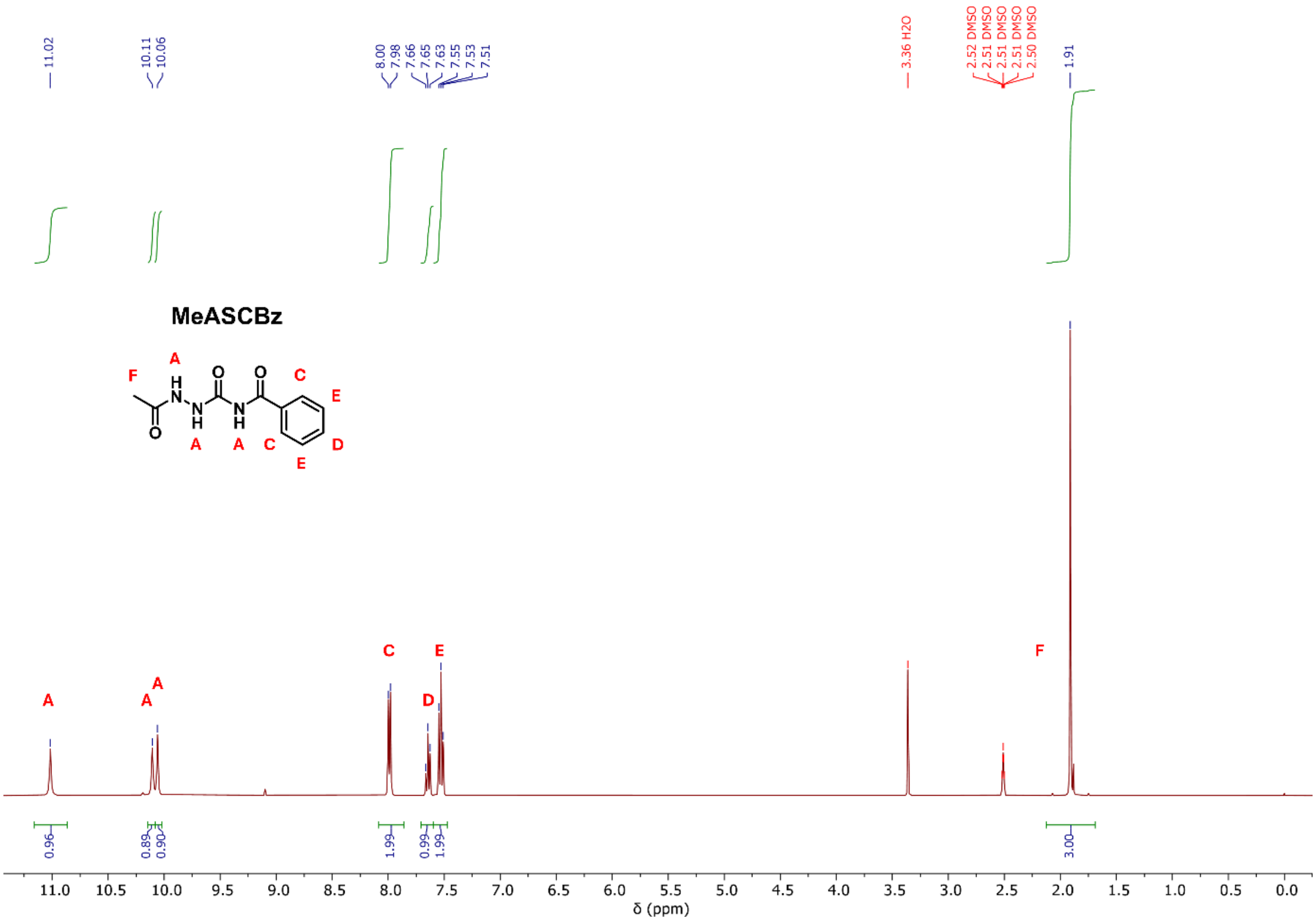


**Figure S14.** $^{1}$H NMR spectrum of **MeASCBz** in DMSO-$d_6$.

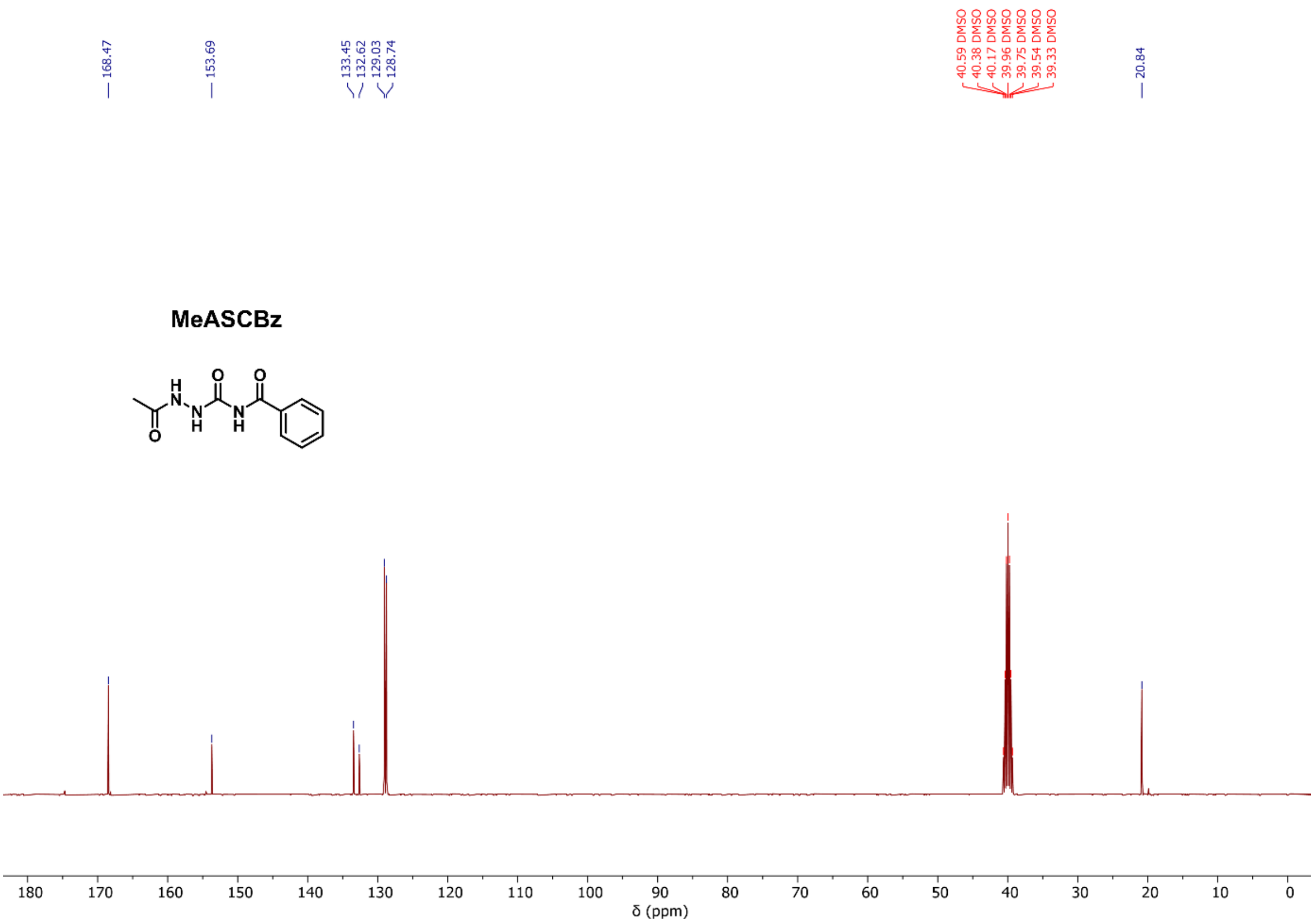


**Figure S15.** $^{13}C$ NMR spectrum of **MeASCBz** in DMSO-$d_6$.

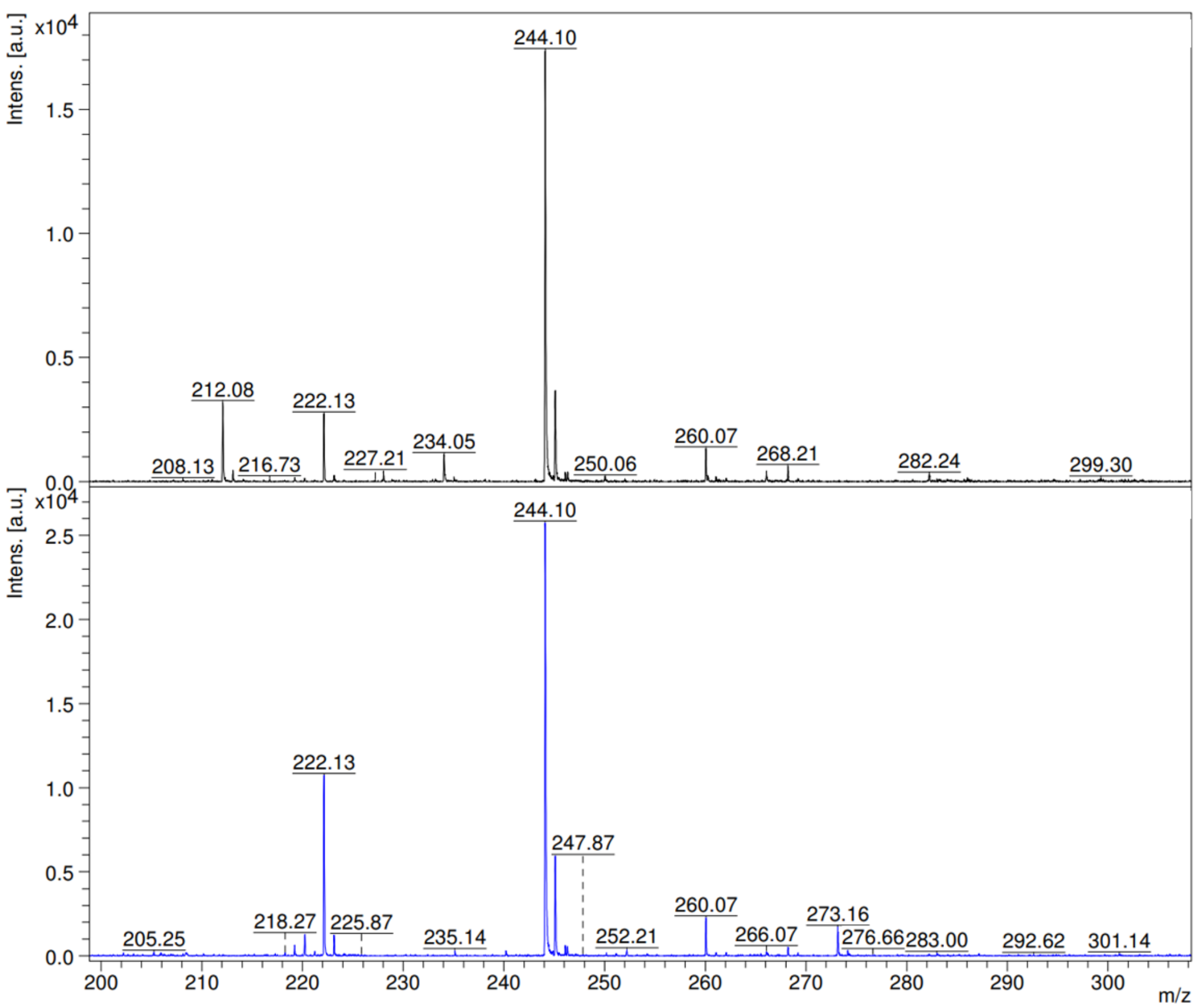


**Figure S16.** MALDI-ToF-MS spectra of **MeASCBz** (black: CHCA, blue: DCTB matrix).

## S5 Cartesian coordinates

The Cartesian coordinates of all computed stationary states and proton affinity geometries are provided in a supplementary .zip folder.

## S6 References


(1) te Velde, G.; Bickelhaupt, F. M.; Baerends, E. J.; Fonseca Guerra, C.; van Gisbergen, S. J. A.; Snijders, J. G.; Ziegler, T. Chemistry with ADF. *J. Comput. Chem.* **2001**, *22* (9), 931–967. https://doi.org/10.1002/jcc.1056.

(2) Baerends, E. J.; Aguirre, N. F.; Austin, N. D.; Autschbach, J.; Bickelhaupt, F. M.; Bulo, R.; Cappelli, C.; van Duin, A. C. T.; Egidi, F.; Fonseca Guerra, C.; Förster, A.; Franchini, M.; Goumans, T. P. M.; Heine, T.; Hellström, M.; Jacob, C. R.; Jensen, L.; Krykunov, M.; van Lenthe, E.; Michalak, A.; Mitoraj, M. M.; Neugebauer, J.; Nicu, V. P.; Philipsen, P.; Ramanantoanina, H.; Rüger, R.; Schreckenbach, G.; Stener, M.; Swart, M.; Thijssen, J. M.; Trnka, T.; Visscher, L.; Yakovlev, A.; van Gisbergen, S. The Amsterdam Modeling Suite. *J. Chem. Phys.* **2025**, *162* (16), 162501. https://doi.org/10.1063/5.0258496.

(3) Fonseca Guerra, C.; Snijders, J. G.; te Velde, G.; Baerends, E. J. Towards an Order-N DFT Method. *Theor. Chem. Acc.* **1998**, *99* (6), 391–403. https://doi.org/10.1007/s002140050353.

(4) Chemistry, S. T. ADF2023.104, 2023. http://www.scm.com (accessed 2024-01-11).

(5) Becke, A. D. Density-Functional Exchange-Energy Approximation with Correct Asymptotic Behavior. *Phys. Rev. A* **9 AD**, *38* (6), 3098–3100. https://doi.org/10.1103/PhysRevA.38.3098.

(6) Lee, C.; Yang, W.; Parr, R. G. Development of the Colle-Salvetti Correlation-Energy Formula into a Functional of the Electron Density. *Phys. Rev. B* **1 AD**, *37* (2), 785–789. https://doi.org/10.1103/PhysRevB.37.785.

(7) Grimme, S.; Antony, J.; Ehrlich, S.; Krieg, H. A Consistent and Accurate Ab Initio Parametrization of Density Functional Dispersion Correction (DFT-D) for the 94 Elements H-Pu. *J. Chem. Phys.* **2010**, *132* (15). https://doi.org/10.1063/1.3382344.

(8) Grimme, S.; Ehrlich, S.; Goerigk, L. Effect of the Damping Function in Dispersion Corrected Density Functional Theory. *J. Comput. Chem.* **2011**, *32* (7), 1456–1465. https://doi.org/10.1002/jcc.21759.

(9) Lenthe, E. van; Baerends, E. J.; Snijders, J. G. Relativistic Regular Two-component Hamiltonians. *J. Chem. Phys.* **1993**, *99* (6), 4597–4610. https://doi.org/10.1063/1.466059.

(10) van Lenthe, E.; Baerends, E. J.; Snijders, J. G. Relativistic Total Energy Using Regular Approximations. *J. Chem. Phys.* **1994**, *101* (11), 9783–9792. https://doi.org/10.1063/1.467943.

(11) Nieuwland, C.; Zaccaria, F.; Fonseca Guerra, C. Understanding Alkali Metal Cation Affinities of Multi-Layer Guanine Quadruplex DNA. *Phys. Chem. Chem. Phys.* **2020**, *22* (37), 21108–21118. https://doi.org/10.1039/d0cp03433a.

(12) Vermeeren, P.; Wolters, L. P.; Paragi, G.; Fonseca Guerra, C. Cooperative Self-Assembly in Linear Chains Based on Halogen Bonds. *ChemPlusChem* **2021**, *86* (6), 812–819. https://doi.org/10.1002/cplu.202100093.

(13) Ahmed, U.; Johansson, M. P.; Lehtola, S.; Sundholm, D. Density Functional Benchmark for Quadruple Hydrogen Bonds. *Phys. Chem. Chem. Phys.* **2025**, *27* (17), 8706–8718. https://doi.org/10.1039/d5cp00836k.

(14) Mitchell, E. C.; Azevedo Santos, L.; Vermeeren, P.; Lahm, M. E.; Fonseca Guerra, C.; Schaefer III, H. F.; Bickelhaupt, F. M. Hydrogen Bond Benchmark: Focal-Point Analysis and Assessment of DFT Functionals. *J. Comput. Chem.* **2025**, *46* (30), e70265. https://doi.org/10.1002/jcc.70265.

(15) Van Lenthe, E.; Baerends, E. J. Optimized Slater-Type Basis Sets for the Elements 1–118. *J. Comput. Chem.* **2003**, *24* (9), 1142–1156. https://doi.org/10.1002/jcc.10255.

(16) Franchini, M.; Philipsen, P. H. T.; van Lenthe, E.; Visscher, L. Accurate Coulomb Potentials for Periodic and Molecular Systems through Density Fitting. *J. Chem. Theory Comput.* **2014**, *10* (5), 1994–2004. https://doi.org/10.1021/ct500172n.

(17) Becke, A. D. A Multicenter Numerical Integration Scheme for Polyatomic Molecules. *J. Chem. Phys.* **1988**, *88* (4), 2547–2553. https://doi.org/10.1063/1.454033.

(18) Pracht, P.; Bohle, F.; Grimme, S. Automated Exploration of the Low-Energy Chemical Space with Fast Quantum Chemical Methods. *Phys. Chem. Chem. Phys.* **2020**, *22* (14), 7169–7192. https://doi.org/10.1039/C9CP06869D.
(19) Klamt, A. Conductor-like Screening Model for Real Solvents: A New Approach to the Quantitative Calculation of Solvation Phenomena. *J. Phys. Chem.* **1995**, *99* (7), 2224–2235. https://doi.org/10.1021/j100007a062.
(20) Klamt, A.; Jonas, V. Treatment of the Outlying Charge in Continuum Solvation Models. *J. Chem. Phys.* **1996**, *105* (22), 9972–9981. https://doi.org/10.1063/1.472829.
(21) Klamt, A.; Schüürmann, G. COSMO: A New Approach to Dielectric Screening in Solvents with Explicit Expressions for the Screening Energy and Its Gradient. *J Chem Soc Perkin Trans 2* **1993**, No. 5, 799–805. https://doi.org/10.1039/p29930000799.
(22) Pye, C. C.; Ziegler, T. An Implementation of the Conductor-like Screening Model of Solvation within the Amsterdam Density Functional Package. *Theor. Chem. Acc. Theory Comput. Model. Theor. Chim. Acta* **1999**, *101* (6), 396–408. https://doi.org/10.1007/s002140050457.
(23) Allinger, N. L.; Zhou, X.; Bergsma, J. Molecular Mechanics Parameters. *J. Mol. Struct. THEOCHEM* **1994**, *312* (1), 69–83. https://doi.org/10.1016/S0166-1280(09)80008-0.
(24) Jacobsen, H.; Bérces, A.; Swerhone, D. P.; Ziegler, T. Analytic Second Derivatives of Molecular Energies: A Density Functional Implementation. *Comput. Phys. Commun.* **1997**, *100* (3), 263–276. https://doi.org/10.1016/S0010-4655(96)00119-1.
(25) Bérces, A.; Dickson, R. M.; Fan, L.; Jacobsen, H.; Swerhone, D.; Ziegler, T. An Implementation of the Coupled Perturbed Kohn-Sham Equations: Perturbation Due to Nuclear Displacements. *Comput. Phys. Commun.* **1997**, *100*, 247–262. https://doi.org/10.1016/s0010-4655(96)00120-8.
(26) Wolff, S. K. Analytical Second Derivatives in the Amsterdam Density Functional Package. *Int. J. Quantum Chem.* **2005**, *104* (5), 645–659. https://doi.org/10.1002/qua.20653.
(27) Legault; Claude. CYLview2.0, 2020. http://www.cylview.org (accessed 2024-07-08).
(28) Mardirossian, N.; Head-Gordon, M. Thirty Years of Density Functional Theory in Computational Chemistry: An Overview and Extensive Assessment of 200 Density Functionals. *Mol. Phys.* **2017**, *115* (19), 2315–2372. https://doi.org/10.1080/00268976.2017.1333644.
(29) Evans, M. G.; Polanyi, M. Some Applications of the Transition State Method to the Calculation of Reaction Velocities, Especially in Solution. *Trans. Faraday Soc.* **1935**, *31* (0), 875–894. https://doi.org/10.1039/TF9353100875.
(30) Van Zeist, W.-J.; Bickelhaupt, F. M. The Activation Strain Model of Chemical Reactivity. *Org. Biomol. Chem.* **2010**, *8* (14), 3118. https://doi.org/10.1039/b926828f.
(31) Vermeeren, P.; Van Der Lubbe, S. C. C.; Fonseca Guerra, C.; Bickelhaupt, F. M.; Hamlin, T. A. Understanding Chemical Reactivity Using the Activation Strain Model. *Nat. Protoc.* **2020**, *15* (2), 649–667. https://doi.org/10.1038/s41596-019-0265-0.
(32) Vermeeren, P.; Hamlin, T. A.; Bickelhaupt, F. M. Chemical Reactivity from an Activation Strain Perspective. *Chem. Commun.* **2021**, *57* (48), 5880–5896. https://doi.org/10.1039/d1cc02042k.
(33) Bickelhaupt, F. M. Understanding Reactivity with Kohn–Sham Molecular Orbital Theory: E2–SN2 Mechanistic Spectrum and Other Concepts. *J. Comput. Chem.* **1999**, *20* (1), 114–128. https://doi.org/10.1002/(SICI)1096-987X(19990115)20:1%253C114::AID-JCC12%253E3.0.CO;2-L.
(34) Bickelhaupt, F. M.; Houk, K. N. Analyzing Reaction Rates with the Distortion/Interaction-Activation Strain Model. *Angew. Chem. Int. Ed.* **2017**, *56* (34), 10070–10086. https://doi.org/10.1002/anie.201701486.
(35) Bickelhaupt, F. M.; Baerends, E. J. Kohn-Sham Density Functional Theory: Predicting and Understanding Chemistry. In *Reviews in Computational Chemistry*; John Wiley & Sons, Inc., 2007; pp 1–86. https://doi.org/10.1002/9780470125922.ch1.
(36) Hamlin, T. A.; Vermeeren, P.; Fonseca Guerra, C.; Bickelhaupt, F. M. 8 Energy Decomposition Analysis in the Context of Quantitative Molecular Orbital Theory. In *Complementary Bonding Analysis*; Simon, G., Ed.; De Gruyter: Berlin, Boston, 2021; pp 199–212. https://doi.org/10.1515/9783110660074-008.
(37) Fu, D.; Pu, W.; Escorihuela, J.; Wang, X.; Wang, Z.; Chen, S.; Sun, S.; Wang, S.; Zuilhof, H.; Xia, H. Acylsemicarbazide Moieties with Dynamic Reversibility and Multiple Hydrogen

Bonding for Transparent, High Modulus, and Malleable Polymers. *Macromolecules* **2020**, *53* (18), 7914–7924. https://doi.org/10.1021/acs.macromol.0c01667.
(38) Maessen, S. J. D.; Lekanne Deprez, S.; Vermeeren, P.; Van Den Bersselaar, B. W. L.; Lutz, M.; Heuts, J. P. A.; Fonseca Guerra, C.; Palmans, A. R. A. Structure–Property Relationships to Direct the Dynamic Properties of Acylsemicarbazide-Based Materials. *Polym. Chem.* **2025**, *16* (3), 290–300. https://doi.org/10.1039/d4py01296h.
(39) International Union of Pure and Applied Chemistry (IUPAC). Transition State Theory. https://doi.org/10.1351/goldbook.T06470.
